# Cyber catalogue and revision of the nematode genus *Enchodelus* (Dorylaimida, Nordiidae)

**DOI:** 10.3897/BDJ.12.e126315

**Published:** 2024-09-19

**Authors:** Milka Elshishka, Aleksandar Mladenov, Stela Altash, Sergio Álvarez-Ortega, Reyes Peña-Santiago, Vlada Peneva

**Affiliations:** 1 Institute of Biodiversity and Ecosystem Research, Bulgarian Academy of Sciences, Sofia, Bulgaria Institute of Biodiversity and Ecosystem Research, Bulgarian Academy of Sciences Sofia Bulgaria; 2 Rey Juan Carlos University, Móstoles, Spain Rey Juan Carlos University Móstoles Spain; 3 University of Jaén, Jaén, Spain University of Jaén Jaén Spain

**Keywords:** collection, databases, distribution, DNA, new species, research infrastructures

## Abstract

**Background:**

The genus *Enchodelus* is an intriguing free-living dorylaimid nematode taxon. Its representatives display a distinct distributional pattern as they are mainly spread in high altitudinal enclaves of the Northern Hemisphere, being often associated with mosses and cliff vegetation. Although their feeding habits have not been studied with experimental protocols, it is traditionally assumed that they are omnivorous.

The genus *Enchodelus* has not been recently revised; descriptions of many ‘old species’ (that have been described long ago and have not been reported since their original discovery) are of poor quality, hardly discoverable and do not conform to the nowadays taxonomical standards. Thus, a comprehensive compilation and analysis of their literature data is indispensable to provide new insights into the taxonomy of the genus and to elucidate its evolutionary relationships.

**New information:**

This contribution provides a cyber catalogue of all *Enchodelus* species, 28 in total. It compiles available information from the key European Research Infrastructures, such as TreatmentBank, Swiss Institute of Bioinformatics Literature Services (SIBiLS), the Catalogue of Life (CoL), Global Biodiversity Information Facility (GBIF), European Nucleotide Archive (ENA) and Biodiversity Literature Repository (BLR). Data about their distribution (geographical records and habitats) are incorporated too and all brought together. It is completed with discussion and notes for some species, along with information on species distributions and microhabitats. Here, all available information on *Enchodelus* species is brought together. This will contribute to a more complete assessment of species diversity and distribution and support further biogeographical and ecological research.

Besides, type material *Enchodelusvestibulifer* Altherr, 1952, deposited in the Museo Cantonale di Storia Naturale di Lugano (Switzerland), is re-examined and the species is considered as *incertae sedis*. Further, a new species of the genus found in Caucasus, Georgia is described after its morphological and molecular study; also morphological and molecular data for *E.macrodorus* (de Man, 1880) Thorne, 1939, the type species of the genus, collected from Spain are provided.

## Introduction

In his monograph devoted to the superfamily Dorylaimoidea, Thorne (1939) erected the genus *Enchodelus* to accommodate five new species, namely *E.arcuatus* Thorne, 1939, *E.brevidentatus* Thorne, 1939, *E.laevis* Thorne, 1939, *E.striatus* Thorne, 1939 and *E.teres* Thorne, 1939, as well as eight transferred from *Dorylaimus* Dujardin, 1845 and *Dorylaimellus* Cobb, 1913, namely *E.analatus* (Ditlevsen, 1927), *E.conicaudatus* (Ditlevsen, 1927), *E.faeroensis* (Ditlevsen, 1928), *E.groenlandicus* (Ditlevsen, 1927), *E.hopedorus* (Thorne, 1929), *E.macrodorus* (de Man, 1880), *E.macrodoroides* (Steiner, 1914) and *E.vesuvianus* (Cobb, 1893), all of them characterised by having double guiding ring, odontophore with developed flanges and diovarian female genital system. *Enchodelusmacrodorus* was proposed as the type species of the new genus.

Several decades later, Ahmad and Jairajpuri (1980) revised the genus and split it into five subgenera (*Enchodelus*, *Heterodorus*, *Nepalus*, *Paraenchodelus* and *Rotundus*), based on some morphological characters, such as lip region shape, odontostyle length, morphology of odontophore, shape of tail and arrangement of ventromedian supplements. At the same time, the authors considered that *Enchodelus* contains two fairly distinct groups of species, one with conoid tails and the other with rounded tails. Subsequently, *Eliava and Eliashvily (1990)* provided a detailed overview of the family Nordiidae and presented the original descriptions and a key for all previously described species of genus *Enchodelus*.

The new millennium brought new relevant contributions to the study of *Enchodelus* diversity: (i) Guerrero and Peňa-Santiago (2007) re-described six species of Thorne’s material: *E.arcuatus*, *E.brevidentatus*, *E.geraldi* Winiszewska-Slipinska, 1987 (= *Enchodelusmacrodoroidesapud* Thorne, 1939) , *E.hopedorus*, *E.macrodorus* and *E.striatus*; (ii) seven new species were described in the period 2008-2012: *E.ameliae* Guerrero, Liebanas & Peña-Santiago, 2008 and *E.longispiculus* Guerrero, Liebanas & Peña-Santiago, 2008 from Spain, *E.babakicus* Pedram, Nicknam, Guerrero, Ye & Robbins, 2009 and *E.sardashtensis* Pedram, Pourjam, Robbins, Ye & Peña-Santiago, 2011 from Iran, *E.parahopedoroides* Ciobanu, Popovici, Guerrero & Peña-Santiago, 2010 and *E.carpaticus* Ciobanu, Popovici, Guerrero & Peña-Santiago, 2010 from Romania and *E.makarovae* Elshishka, Lazarova & Peneva, 2012 from the Russian Arctic; (iii) Andrássy (2009a) and Andrássy (2009b) revised the taxonomy of *Enchodelus* species, retrieving the genus *Heterodorus* (= *Nepalus*, *Paraenchodelus*) as a valid genus to include those species displaying conical tail and few ventromedian supplements with hiatus and retaining under *Enchodelus* (= *Rotundus*) the rounded-tailed forms with ventromedian supplements without hiatus; (iv) molecular data (Pedram et al. 2009, Pedram et al. 2011, Pedram et al. 2015) supported the opinion of Andrássy that the species with rounded and conical tail form two different groups (clades); (v) Peña-Santiago (2021b) provided the list of the species of genus *Enchodelus* with their synonyms and geographical records.

The genus *Enchodelus* currently includes 28 species, which are typical components of septentrional (Northern Hemisphere) fauna (Peña-Santiago 2021a), with the exception of *E.brasiliensis* Meyl, 1957, only known to occur in Brazil. The members of the genus inhabit high altitudes (1260-4400 m a.s.l.) and latitudes (northern territories), frequently associated with mosses and rock vegetation (Ahmad and Jairajpuri 1980, Eliava and Eliashvily 1990, Peneva et al. 2009, Elshishka et al. 2012). As the species are found mainly in natural habitats, the genus can be considered to have a conservation value.

No recent revision of *Enchodelus* members has hitherto been published. Descriptions of many ‘old species’ are of poor quality, hardly discoverable and do not conform to the nowadays taxonomical standards. Actually, information available from databases often is limited to some of the species and usually incomplete as relevant data are missing. Consequently, a comprehensive compilation and analysis of literature data is indispensable to reach new insights into the taxonomy of the genus and to elucidate its evolutionary relationships. Thus, this contribution aims to provide a cyber catalogue of *Enchodelus* species, where all available data for the species are accessible and collected in one place which will greatly facilitate future research. Moreover, a re-examination of type material of *Enchodelusvestibulifer* Altherr, 1952, deposited in the Museo Cantonale di Storia Naturale di Lugano (Switzerland), is also presented, as well as the description of a new species of the genus found in Caucasus, Georgia after its morphological and molecular study and also morphological and molecular data for *E.macrodorus*, collected from Spain.

## Materials and methods

The cyber catalogue compiles all kinds of data (taxonomical, sequences, names, records, tables, figures) provided in some of European Research Infrastructures, such as TreatmentBank, Swiss Institute of Bioinformatics Literature Services (SIBiLS), the Catalogue of Life (CoL), Global Biodiversity Information Facility (GBIF), European Nucleotide Archive (ENA) and Biodiversity Literature Repository (BLR) for the species of the genus *Enchodelus*. This information is completed with records, both geographical and ecological (habitats) and notes or discussion about them. Habitats are reported as in the original paper, with the scientific names of the plants adapted according to their current systematics.

The new species described was collected by one of authors (V. Peneva) from moss on stone (*Tortellasquarrosa* (Brid.) Limpr.) in Caucasus, Georgia. Nematodes were extracted by using the Baermann funnel method (van Bezooijen 2006) for 48 hours exposition, killed by gentle heat and fixed in 4% formalin. Specimens were processed in anhydrous glycerine (Seinhorst 1959) and mounted on permanent slides.

**Figure 1.**
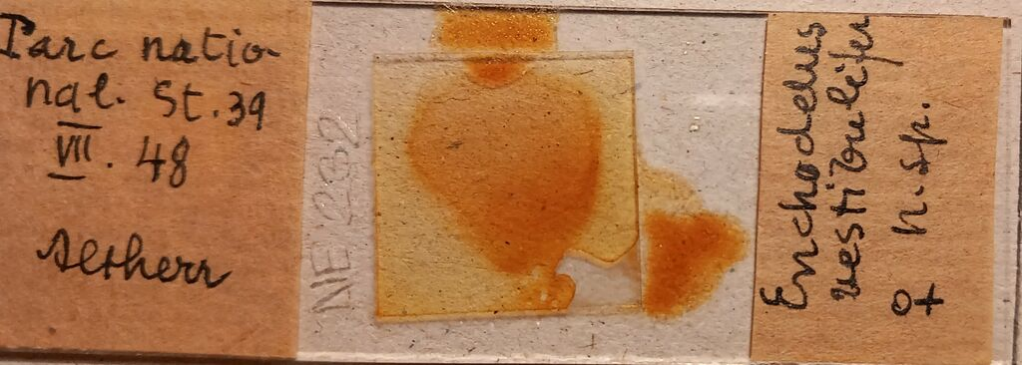
Slide with original label: *Enchodelusvestibulifer*.

Type material of *E.vestibulifer*, belonging to Edmond Altherr’s collection, deposited at the Museo Cantonale di Storia Naturale di Lugano (Switzerland), is re-examined. The single female specimen is preserved in one permanent glycerine slide, in poor condition. The information included on the label is presented in the catalogue and in Fig. 1.

A few specimens of *E.macrodorus* were found in soil samples from grass in Spain. Nematodes were extracted from soil samples by sieving and the sucrose-centrifugation technique (specific gravity = 1.18), following the protocol of California Department of Food and Agriculture (CDFA 2015; based on Jenkins (1964)), killed by gentle heat and fixed in 4% formalin.

Drawings were prepared using an Olympus BX 51 compound microscope with a drawing tube. Photographs were taken using an Axio Imager.M2-Carl Zeiss compound microscope equipped with a digital camera (ProgRes C7) and specialised software (CapturePro Software 2.8). Measurements were made using an Olympus BX 41 light microscope with a drawing tube and digitising tablet (CalComp Drawing Board III, GTCO CalCom Peripherals, Scottsdale, AZ, USA) and Digitrak 1.0f computer programme (Philip Smith, John Hutton Institute, Dundee, UK). Terminology was adopted according to Peña-Santiago (2021a). The locations of pharyngeal gland nuclei are given following Loof and Coomans (1970) and Andrássy (1998).

**DNA isolation, PCR and sequencing**

The specimen intended for the molecular study was identified on temporary mounts; a standard set of photomicrographs was taken. Genomic DNA (gDNA) was isolated using 5% suspension of deionised water and Chelex®, containing 0.1 mg/ml proteinase K; samples were incubated at 56°C or overnight, boiled at 90°C for 8 min and centrifuged at 14,000´g for 10 min. A genetic marker sequenced was the large (28S) ribosomal subunit RNA coding regions. Partial fragments of the 28S rRNA gene (domains D1-D3; ~ 1000 bp) were amplified using the forward primer LSU5 (5'-TAG GTC GAC CCG CTG AAY TTA AGC A-3') (Littlewood et al. 2000) and the reverse primer 1500R (5'-GCT ATC CTG AGG GAA ACT TCG-3') (Tkach et al. 1999). PCR amplifications were performed with 2´ MyFi™ DNA Polymerase mix (Bioline Inc., Taunton, USA; Cat. # BIO-25049) in a total volume of 20 μl, containing 8 pmol of each primer and ca. 50 ng of gDNA. The amplification profile for 28S rDNA comprised an initial denaturation at 94°C for 3 min followed by 40 cycles (30 s at 94°C; 30 s at 55°C; and 2 min at 72°C) and a final extension step at 72°C for 7 min. PCR amplicons were purified and sequenced directly for both strands using the PCR primers at Macrogen Europe (Amsterdam, the Netherlands). Contiguous sequences were assembled, quality checked and edited manually using MEGA7 (Kumar et al. 2016) and subjected to a BLASTn search on the NCBI GenBank database. The newly-obtained sequences were submitted to the GenBank database under accession numbers: PP662485, for *Enchodelusenguriensis* sp. nov. - 28S; PP662484, for *E.macrodorus* - 28S; and PP657694, for *E.macrodorus* - 18S.

**Phylogenetic and sequence analysis**

The newly-obtained sequences were aligned with another fifty-two D2–D3 expansion segments of 28S rRNA gene sequences available in GenBank using ClustalX 1.83 (Chenna 2003). Outgroup taxa were chosen, based on previously-published data (Álvarez‐Ortega and Peña‐Santiago 2019, Álvarez-Ortega 2020). The alignment was analysed with Bayesian Inference (BI) at the CIPRES Science Gateway (Miller et al. 2010), using MrBayes 3.2.7a (Ronquist et al. 2012). The best fit model of DNA evolution was obtained using jModelTest 2.1.10 (Darriba et al. 2012) with the Akaike Information Criterion (AIC). The Akaike-supported model, the base frequency, the proportion of invariable sites and the gamma distribution shape parameters and substitution rates in the AIC were then used in phylogenetic analyses. BI analysis under the general time reversible model with a proportion of invariable sites and a gamma-shaped distribution (GTR+I+G) was initiated with a random starting tree and run with the four Metropolis-coupled Markov Chain Monte Carlo (MCMC) for 2 x 10^6^ generations. The topologies were used to generate a 50% majority rule consensus tree. Posterior probabilities (PP) over 70% are given on appropriate clades. The trees were visualised with the programme FigTree v.1.4.3 and drawn with Adobe Illustrator CC.

## Taxon treatments

### Enchodelus

Thorne, 1939

3CA9F173-4978-5B1B-A012-C375D28E03C5

907FC242-EF1E-4072-AF46-FD5BBB4A13D4

https://www.catalogueoflife.org/data/taxon/9CKPN

https://www.gbif.org/species/2282676

https://sibils.text-analytics.ch/search/?query=enchodelus&tab=plazi#results-section

Enchodelus (*Enchodelus* Thorne, 1939); Ahmad and Jairajpuri (1980):12-13 [diagnosis; key to species] BLR: https://doi.org/10.5281/zenodo.8144934
Enchodelus (*Enchodelus* Thorne, 1939); Jairajpuri and Ahmad (1992);179 [diagnosis; list] BLR:https://doi.org/10.5281/zenodo.10849482
Enchodelus (*Rotundus* Ahmad & Jairajpuri, 1980); Ahmad and Jairajpuri (1980):13-14 [diagnosis; key to species] BLR: https://doi.org/10.5281/zenodo.8144936
Enchodelus (*Rotundus* Ahmad & Jairajpuri, 1980); Jairajpuri and Ahmad (1992):183 [diagnosis; list] BLR: https://doi.org/10.5281/zenodo.10849492
Enchodelus Thorne, 1939; Thorne (1939):61-62 [diagnosis; key to species] BLR: https://doi.org/10.5281/zenodo.11003096
Enchodelus Thorne, 1939; Goodey (1951):298, 300-301 [diagnosis; list] BLR: https://doi.org/10.5281/zenodo.11013374
Enchodelus Thorne, 1939; Andrássy (1958b):324 [diagnosis] BLR: https:// doi.org/10.5281/zenodo.10842858
Enchodelus Thorne, 1939; Goodey (1963):441-443 [diagnosis; list] BLR: https://doi.org/10.5281/zenodo.10891552
Enchodelus Thorne, 1939; Zullini (1973):406-408 [list; key to species] BLR: https://doi.org/10.5281/zenodo.10845261
Enchodelus Thorne, 1939; Baqri and Jairajpuri (1974):145-146 [key to species] BLR: https://doi.org/10.5281/zenodo.8117936
Enchodelus Thorne, 1939; Bongers (1988):324 [diagnosis] BLR: https://doi.org/10.5281/zenodo.10822196
Enchodelus Thorne, 1939; Eliava and Eliashvily (1990)BLR: https://doi.org/10.5281/zenodo.11003031
Enchodelus Thorne, 1939; Jairajpuri and Ahmad (1992):178-183 [diagnosis; key to subgenera] BLR: https://doi.org/10.5281/zenodo.10849480
Enchodelus Thorne, 1939; Loof (1999):86 [diagnosis] BLR: https://doi.org/10.5281/zenodo.11003046
Enchodelus Thorne, 1939; Vinciguerra (2006):457-458 [diagnosis, figure, list]
Enchodelus Thorne, 1939; Guerrero et al. (2008a):727, 730-732 [compendium of species of *Enchodelushopedorus* group; key to species of *Enchodelushopedorus* group]
Enchodelus Thorne, 1939; Guerrero et al. (2008b):467-468 [compendium of species of *Enchodelusmacrodorus* group; key to species of *Enchodelusmacrodorus* group]
Enchodelus Thorne, 1939; Andrássy (2009b):382-385 [diagnosis; list; key to European species] BLR: https://doi.org/10.5281/zenodo.10821943
Enchodelus Thorne, 1939; Elshishka et al. (2012):21 [key to species of *Enchodelusmacrodorus* group]
Enchodelus Thorne, 1939; Peña-Santiago (2021b):205-212 [list; geographical records]
Enchodelus Thorne, 1939; Hodda (2022):28 [list]

#### Diagnosis

Nordiidae, Pungentinae. Small- to medium-sized nematodes, 0.6-2.5 mm long. Cuticle dorylamoid. Lip region offset by depression or constriction, with variably amalgamate lips. Amphid fovea cup-like, its aperture occupying ca. one-half of lip region diameter. Odontostyle slender, with narrow lumen and small aperture, longer (1-2 times) than lip region diameter. Guiding ring double, low. Odontophore with developed basal flanges. Pharynx entirely muscular, gradually enlarging into a pharyngeal expansion occupying less than one-half of total neck length, with S_2_N as large as DN and rather anterior in position. Female genital system diovarian, with long and tripartite uterus, distinct *pars refringens vaginae* and transverse vulva. Tail similar in sexes, short and rounded to convex conoid. Spicules dorylaimid. Ventromedian supplements 7-18 in number, spaced, without hiatus.

Type species: *Enchodelusmacrodorus* (de Man, 1880) Thorne, 1939

### Enchodelus macrodorus

(de Man, 1880) Thorne, 1939

D8314892-5951-51C8-BE01-FCCF871E00C4

7CAD1A7A-726F-4CF0-85F2-B0B5E1B24EB7

https://www.catalogueoflife.org/data/taxon/886TQ

https://www.gbif.org/species/2282680

https://www.ebi.ac.uk/ena/browser/view/Taxon:289013

https://sibils.text-analytics.ch/search/?query=enchodelus%20macrodorus#results-section

Dorylaimus macrodorus de Man, 1880; de Man (1880) BLR: https://doi.org/10.5281/zenodo.10852798
Dorylaimus macrodorus ; de Man (1884) BLR: https://doi.org/10.5281/zenodo.10867883
Dorylaimus macrodorus ; de Man (1912):454-456 BLR: https://doi.org/10.5281/zenodo.11003008
Dorylaimus macrodorus ; Menzel (1913) BLR: https://doi.org/10.5281/zenodo.10797350
Dorylaimus macrodorus ; Brakenhoff (1914) BLR: https://doi.org/10.5281/zenodo.10822895
Dorylaimus macrodorus ; Steiner (1914):262 [list] BLR: https://doi.org/10.5281/zenodo.11003080
Dorylaimus macrodorus ; Hofmänner and Menzel (1915):186-187 BLR: https://doi.org/10.5281/zenodo.11003145
Dorylaimus macrodorus ; Steiner (1916a):69-70 BLR: https://doi.org/10.5281/zenodo.11003157
Dorylaimus macrodorus ; Steiner (1916b):345 BLR: https://doi.org/10.5281/zenodo.11003151
Dorylaimus macrodorus ; Micoletzky (1921) BLR: https://doi.org/10.5281/zenodo.10932729
Dorylaimus macrodorus ; Schneider (1923) BLR: https://doi.org/10.5281/zenodo.10795142
Dorylaimus macrodorus ; Kreis (1924) BLR: https://doi.org/10.5281/zenodo.10892094
Dorylaimus macrodorus ; Stefanski (1924):55-57
Dorylaimus macrodorus ; Micoletzky (1925):181-182
Dorylaimus macrodorus ; Rahm (1925):182
Dorylaimus macrodorus ; Stefanski (1927) BLR:https://doi.org/10.5281/zenodo.10941762
Dorylaimus macrodorus ; Soós (1936):61 [list] BLR:https://doi.org/10.5281/zenodo.10944890
Dorylaimus macrodorus ; Allgén (1953) BLR: https://doi.org/10.5281/zenodo.10809558
Dorylaimus (Doryllium) macrodorus de Man, 1880; Seidenschwarz (1923):42
Dorylaimus (Doryllium) macrodorus de Man, 1880; Allgén (1925) BLR: https://doi.org/10.5281/zenodo.10998093
Dorylaimus (Doryllium) macrodorus de Man, 1880 (Ditlevsen, 1928); Ditlevsen (1928):9
Dorylaimellus macrodorus (de Man, 1880) Thorne & Swanger, 1936; Thorne and Swanger (1936) BLR:https://doi.org/10.5281/zenodo.10845809
Dorylaimellus macrodorus ; *Altherr (1950)* BLR: https://doi.org/10.5281/zenodo.10810136
Dorylaimellus macrodorus ; Altherr (1952):342
Dorylaimellus macrodorus ; Andrássy (1952) BLR: https://doi.org/10.5281/zenodo.11012991
Dorylaimus (Dorylaimellus) macrodorus de Man, 1880 (Thorne & Swanger, 1936): Thorne and Swanger (1936) BLR: https://doi.org/10.5281/zenodo.10845809
Dorylaimus (Dorylaimellus) macrodorus ; Schneider (1939) BLR:https://doi.org/10.5281/zenodo.11028113
Enchodelus (Enchodelus) macrodorus (de Man, 1880) Thorne, 1939
Enchodelus (Enchodelus) macrodorus ; Ahmad and Jairajpuri (1980) BLR: https://doi.org/10.5281/zenodo.8144942
Enchodelus (Enchodelus) macrodorus ; Jairajpuri and Ahmad (1992): 180 [figure] BLR: https://doi.org/10.5281/zenodo.10850166
Enchodelus (Enchodelus) macrodorus ; Choi et al. (1997) BLR: https:// doi.org/10.5281/zenodo.10822472
Enchodelus macrodorus (de Man, 1880) Thorne, 1939; Thorne (1939) BLR:https://doi.org/10.5281/zenodo.11003121
Enchodelus macrodorus ; Goodey (1951) BLR: https://doi.org/10.5281/zenodo.11013374
Enchodelus macrodorus ; Altherr (1953) BLR: https://doi.org/10.5281/zenodo.11012949
Enchodelus macrodorus ; *Andrássy (1958b)* BLR: https://doi.org/10.5281/zenodo.10842858
Enchodelus macrodorus ; Meyl (1961) BLR: https://doi.org/10.5281/zenodo.11015551
Enchodelus macrodorus ; van Rossen and Loof (1962) BLR: https://doi.org/10.5281/zenodo.10845171
Enchodelus macrodorus ; Goodey (1963) BLR: https://doi.org/10.5281/zenodo.10891552
Enchodelus macrodorus ; Jairajpuri and Loof (1967) BLR:https://doi.org/10.5281/zenodo.8122568
Enchodelus macrodorus ; Loof and Coomans (1970) BLR: https://doi.org/10.5281/zenodo.11014428
Enchodelus macrodorus ; Zullini (1970) BLR: https://doi.org/10.5281/zenodo.10850512
Enchodelus macrodorus ; Loof (1971) BRL: hhttps://doi.org/10.5281/zenodo.8152932
Enchodelus macrodorus ; Thorne (1974) BLR: https://doi.org/10.5281/zenodo.10819174
Enchodelus macrodorus ; *Andrássy (1978)*:116 [list]
Enchodelus macrodorus ; Nesterov (1979) BLR: https://doi.org/10.5281/zenodo.11015718
Enchodelus macrodorus ; Vinciguerra (1984) BLR: https://doi.org/10.5281/zenodo.10818036
Enchodelus macrodorus ; Winiszewska-Slipinska (1987) BLR: https://doi.org/10.5281/zenodo.10854940
Enchodelus macrodorus ; Bongers (1988) BLR: https://doi.org/10.5281/zenodo.10822198
Enchodelus macrodorus ; Eliava and Eliashvily (1990):50-51,96 BLR:https://doi.org/10.5281/zenodo.11003031
Enchodelus macrodorus ; Gerber (1991) BLR: https://doi.org/10.5281/zenodo.10883357
Enchodelus macrodorus ; *Nasira et al. (1992)* BLR: https://doi.org/10.5281/zenodo.8122525
Enchodelus macrodorus ; *Popovici (1995a)* BLR: https://doi.org/10.5281/zenodo.8125537
Enchodelus macrodorus ; Loof (1999) BLR: https://doi.org/10.5281/zenodo.11003046
Enchodelus macrodorus ; Ahmad et al. (2002) BLR: https://doi.org/10.5281/zenodo.10683060
Enchodelus macrodorus ; Poiras (2006):93 [list] BLR: https://doi.org/10.5281/zenodo.10717726
Enchodelus macrodorus ; Guerrero and Peňa-Santiago (2007) BLR: https://doi.org/10.5281/zenodo.8111782
Enchodelus macrodorus ; Guerrero et al. (2008b) BLR: https://doi.org/10.5281/zenodo.8111849
Enchodelus macrodorus ; Pedram et al. (2009) BLR: https://doi.org/10.5281/zenodo.8114781
Enchodelus macrodorus ; Andrássy (2009b)BLR: https://doi.org/10.5281/zenodo.10821949
Enchodelus macrodorus ; Ciobanu et al. (2010a) BLR:https://doi.org/10.5281/zenodo.8111705;
Enchodelus macrodorus ; Holovachov (2014):22 [list] Plazi treatment
Enchodelus macrodorus ; Shahina et al. (2019):223 [list] BLR: https://doi.org/10.5281/zenodo.10710707
Enchodelus macrodorus ; *Peña-Santiago (2021b)*:208-210 BLR: https://doi.org/10.5281/zenodo.11191905

#### Distribution

A typical member of Palearctic nematode fauna, recorded in a myriad of countries and habitats: **Netherlands** (type habitat: moist soil in meadows and marshes - de Man (1880), de Man (1884) /sandy soil with moss - de Man (1912), Loof and Oostenbrink (1962), Loof and Coomans (1970) Loof and Coomans (1970), Bongers (1988)), **Alps** (moss - Franz (1942), Franz and Gunhold (1954)), **Austria** (moss *Hypnumcupressiforme* Hedw - Steiner (1916b), Micoletzky (1921) / moss - Seidenschwarz (1923)), **Bulgaria** (moss - Andrássy (1958a), Katalan-Gateva (1968)), **China** (grassland and shrubs - Ahmad et al. (2002)), **Czech Republic** (meadow, grassland - Háněl (1998)), **Denmark** (moor - Micoletzky (1925)), **Faroe Islands** (Ditlevsen (1928)), **Germany** (river bank - Brakenhoff (1914) / moss - Schneider (1923), Rahm (1925), Schneider (1939) / grassland - Diedrich et al. (1998) / lake, 1136 m a.s.l. - Michiels and Traunspurger (2005)), **Hungary** (moss - Soós (1936), Soós (1940a), Soós (1940b), Andrássy (1952), Andrássy (1958b) / moss, grass, pine - Andrássy (1996), Andrássy (2009b)), **Iran** (grasslands - Pedram et al. (2009), Jabbari et al. (2019)), **Italy** (moss - Zullini (1970) / moss - Vinciguerra (1984)), **Korea** (Betulaplatyphyllavar.japonica Hara - Choi et al. (1997)), **Moldova** (deciduous forests - Poiras (2006)), **Norway** (Jan Mayen Island - Allgén (1953) / Spitzbergen, grasses - Loof (1971)), **Pakistan** (soil around roots of weeds and grasses - Nasira et al. (1992) / freshwater - Nasira et al. (2016) / freshwater - Nasira and Shamim (2018)), **Poland** (moss - Stefanski (1924),Brzeski (1963b) / *Arrhenatherum* sp. - Winiszewska-Slipinska (1987) / primeval forest - Brzeski and Winiszewska-Slipinska (1996)), **Romania** (freshwater - Stefanski (1926), Stefanski (1927), Popovici (1993), Popovici (1995a) / hornbeam-beech forest, 400 m a.s.l - Popovici (1995b), Popovici and Ciobanu (1997) / grasslands - Popovici (1998), Ciobanu and Popovici (2001) / hornbeam-beech forest, grasslands, cliff vegetation, 400-1250 m a.s.l. - Ciobanu et al. (2010a) /grass - Ciobanu and Popovici (2017)), **Russia** (Arctic - Novaya Zemlya Archipelago - Steiner (1916a) / Arctic tundra - Kuzmin (1973), Kuzmin and Gagarin (1990) / Novaya Zemlya Archipelago - Gagarin (1997), Gagarin (2001)/ polar desert - cape Cheluyskin - Chernov et al. (1979), Soloveva et al. (1976)), **Slovakia** (moss - Koniar (1957) / corn - Sabova et al. (1979), Šály (1979) / vineyard - Liskova (1980) / *Alnusglutinosa* Gaertn. - Šály (1980) / *Fagussylvatica* L., *Abiesalba* Mill, *Piceaabies* H.Karst., meadow, moss, riverbank, brook, waterfall - Šály (1985) / *Pinusmugo* Turra, *P.abies* - Liskova et al. (1996) / meadow, river bank - Liskova and Čerevkova (2005) / grassland - Čerevková (2006) / grasslands - Čerevková (2008) / *Lariceto*-*Picetum* - Čerevková and Renčo (2009) / moss, forests, grasslands, potato, cereal, vineyard, riverbank - Liskova and Čerevkova (2011) / spruce forests - Čerevková et al. (2013) / *P.abies*, *Pinussylvestris* L., *Quercusrobur* L., *Acerpseudoplatanus* L., *F.sylvatica* - Renčo (2013) / maize - Čerevková and Cagan (2015) / alpine meadows, 1763-1994 m a.s.l. - Háněl 2017 / *P.abies*, *Larixdecidua* - Renčo and Čerevková (2017) / deciduous forest, wetland, *Fallopiajaponica* (Houtt.), 386-455 m a.s.l. - Čerevková et al. (2019) / *Asclepiassyriaca* L., grassland - Jurová et al. (2020) / deciduous forests, grassland, *Solidagogigantea* Ait. - Čerevková et al. (2020)), **Spain** (Palomo (1979) / wet meadows, soil around poplar, 1400-2925 m a.s.l. - Guerrero et al. (2008b)), **Sweden** (moss - Allgén (1925), van Rossen and Loof (1962)), **Switzerland** (Alps, 2000-4000 m a.s.l. - Menzel (1913), Steiner (1914), Hofmänner and Menzel (1915) / lake with the melting snow water - Kreis (1924) / alpine meadow with *Alchemilla* sp., *Nardusstricta* L., *Trifolium* sp., pine forest, 1900-2200 m a.s.l. - Altherr (1950), Altherr (1952), Altherr (1953)), **UK** (moor, grassland - Banage (1962), Loof and Coomans (1970) / grasslands, 110-160 m a.s.l. - Hodda and Wanless (1994) /spruce forests, 200 m a.s.l. - Ruess et al. (1996)), **Ukraine** (grasses - Nesterov (1979)) and **Uzbekistan** (rice, bean, cotton - Tulaganov (1958) / *Cucumissativus* L. - Tukhtasinov (2023)).

Very sporadically, this species was also recorded in Nearctic (**USA**, *Sporoboluscompositus* (Poir.) Merr. - Orr and Dickerson (1967) / mountain soil - Thorne (1974)) and Indomalayan (**India**, soil near roots of apple - Jairajpuri and Loof (1967) / *Pinuspinea* L. - Ahmad and Jairajpuri (1980)) enclaves.

#### Taxon discussion

*Enchodelusmacrodorus* was originally described by de Man (1880) from the Netherlands. It is the most widely spread *Enchodelus* species. Guerrero and Peňa-Santiago (2007) and Guerrero et al. (2008b) re-described American specimens of *E.macrodorus* studied by Thorne (1939), noted that these specimens apparently fitted very well with those described by de Man (1880), considered that many subsequent records of the species were doubtful or might correspond to other species, provided an emended diagnosis of this species and regarded the populations reported by Thorne (1939), Jairajpuri and Loof (1967), Ahmad and Jairajpuri (1980), Nasira et al. (1992) and Ahmad et al. (2002), also three of the populations reported by Popovici (1995a) to be conspecific with *E.macrodorus*. Later Pedram et al. (2009) reported the species for the first time from Iran and presented the first integrative study of the species.

#### Notes

##### Morphological characterisation

Material examined. One female in good condition, mounted on one slide and collected from Rasquilla, Navalsauz, Avila Province, Spain, in grassland riparian zone close to the Alberche River (N 40º 24.350' W 05º 01.817', elevation 1247 m a.s.l.) in May 2021.

**Figure 2.**
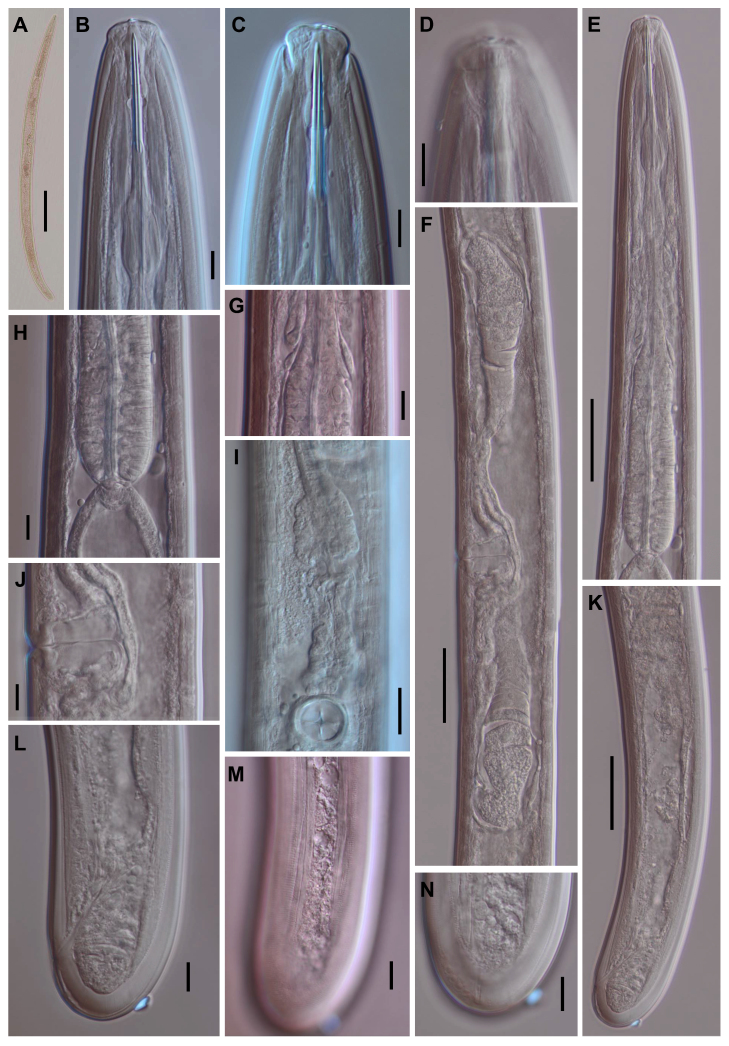
*Enchodelusmacrodorus* (de Man, 1880) Thorne, 1939, female. **A** Entire body; **B-D** Anterior region; **E** Neck; **F** Genital system; **G** Pharyngeal dorsal nuclei; **H** Pharyngeal intestinal junction; **I** Uterus and oviduct junction; **J** Vagina; **K** Posterior body region; **L-N** Tail ends (M detail of the lateral chord ending; N detail of the saccate bodies of tail). Scale bars: **A** 200 µm; **B-D, G, H, J, L-N** 10 µm; **E, F, K** 50 µm; **I** 20 µm.

Measurements: Table 1, Fig. 2

Diagnosis (based in examined specimen)

*Enchodelusmacrodorus* is characterised by its 1.44 mm long body, lip region 18.5 μm wide and offset by weak depression, odontostyle 39.0 μm long or 2.1 times lip region diameter, odontophore flanged and 1.1 times as long as odontostyle, neck 326 μm long, pharyngeal expansion occupying two-fifths of the total neck length, female genital system diovarian, uterus short (63, 68 μm long), *pars refringens vaginae* with two rectangular sclerotisations, vulva (V = 44%), tail short and rounded (20 μm, c = 71, c’ = 0.5) bearing saccate bodies.

Remarks

The studied female perfectly fits, morphologically and morphometrically, with the previous descriptions of this species, especially with the re-description of the Thorne material provided by Guerrero and Peňa-Santiago (2007).

##### Molecular characterisation

**Figure 3.**
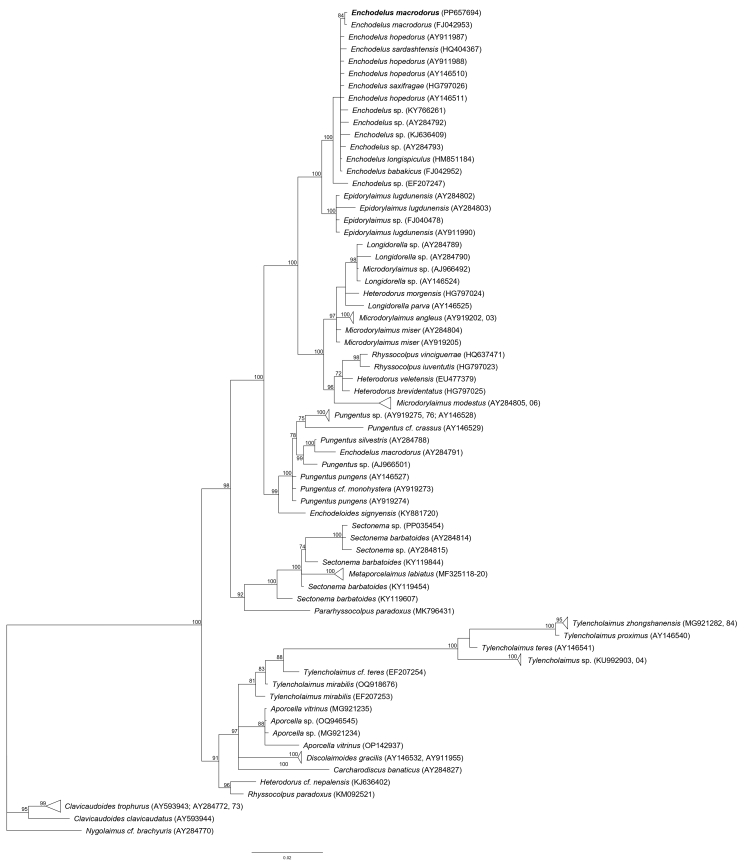
Bayesian 50% majority rule consensus tree as inferred from the partial 18S rRNA gene sequence alignment under the GTR+I+G model. Posterior probability values more than 70% are given on appropriate clades. New sequences are indicated by bold letters.

**Figure 4.**
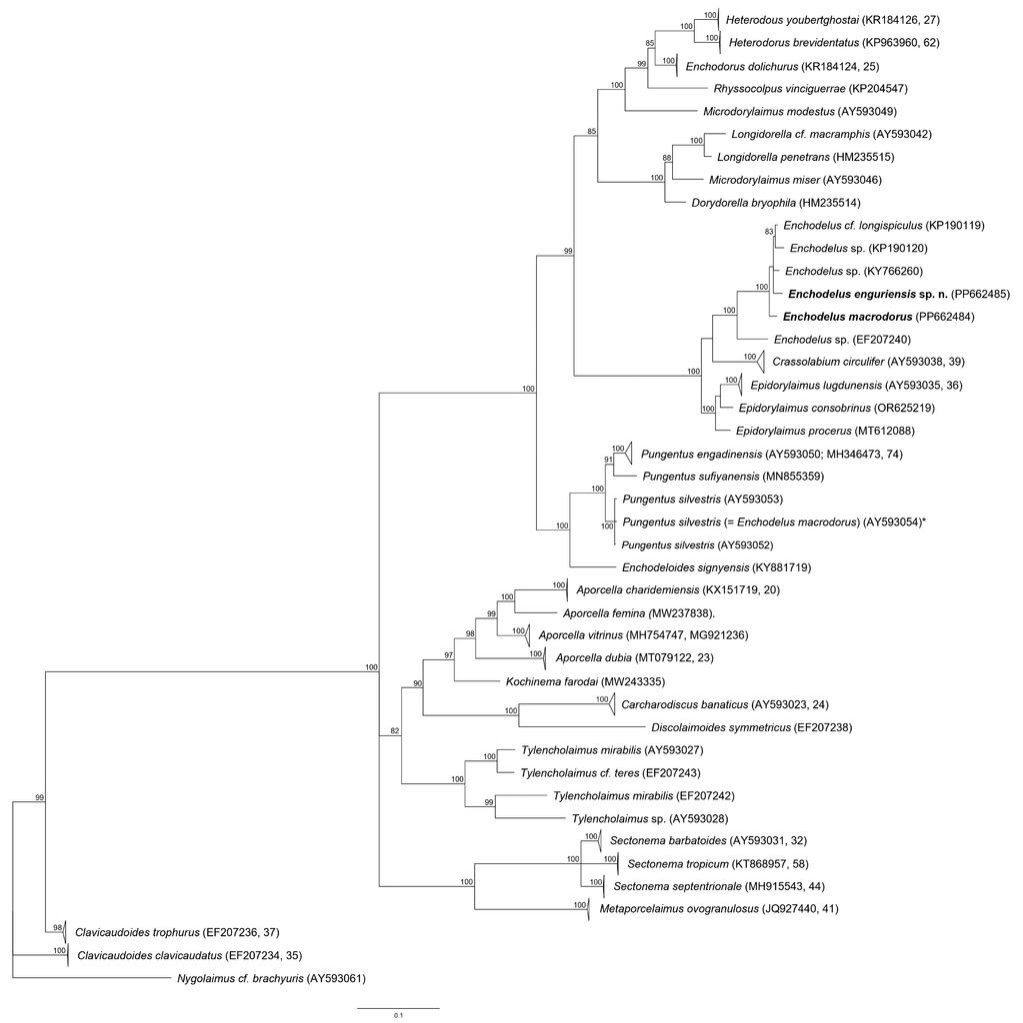
Bayesian 50% majority rule consensus tree as inferred from the D2-D3 expansion segments of 28S rRNA gene sequence alignment under the GTR+I+G model. Posterior probability values more than 70% are given on appropriate clades. New sequences are indicated by bold letters.

One partial 18S rRNA and one D2-D3 of 28S rRNA gene sequences were obtained for this species. In the partial 18S rRNA (Fig. 3) gene tree, the sequence of the Spanish *E.macrodorus* clustered together to another sequence of the same species (FJ042953) and inside of a highly-supported clade (PP = 100) with other *Enchodelus* spp. In the D2-D3 of 28S rRNA (Fig. 4) gene tree, the sequence of the Spanish *E.macrodorus* formed a highly-supported clade (PP = 100) with *E.enguriensis* sp. nov. and another three *Enchodelus* sequences from GenBank.

Regarding the molecular data for this species, it appears that the sequence AY593054 available in GenBank was incorrectly identified or labelled. This sequence is herein identified as belonging to *Pungentussilvestris* (Figs 3, 4). Besides, the sequence AY284791, available in GenBank, was incorrectly identified too and most probably belongs to a *Pungentus* species.

### Enchodelus altherri

Vinciguerra & De Francisci, 1973

E026F6F2-BDE0-55EA-8223-AD9B014C5C23

9776ABF8-69E3-462C-A47F-0001DD75B082

https://www.catalogueoflife.org/data/taxon/9HMJX

https://www.gbif.org/species/12242420

https://sibils.text-analytics.ch/search/?query=enchodelus%20altherri#results-section

Enchodelus (Enchodelus) altherri Vinciguerra & De Francisci, 1973 (Ahmad & Jairajpuri, 1980); Ahmad and Jairajpuri (1980) BLR: https://doi.org/10.5281/zenodo.8144934
Enchodelus altherri Vinciguerra & De Francisci, 1973; Vinciguerra and De Francisci (1973) BLR: https://doi.org/10.5281/zenodo.8143853
Enchodelus altherri ; *Eliava and Eliashvily (1990)*:51,96 BLR: https://doi.org/10.5281/zenodo.11003031
Enchodelus altherri ; *Andrássy (2009b)* BLR: https://doi.org/10.5281/zenodo.10821947
Enchodelus altherri ; *Peña-Santiago (2021b)*:206 BLR: https://doi.org/10.5281/zenodo.11191877

#### Distribution

A typical representative of Palearctic nematode fauna, distributed only in Europe: **Italy** (type habitat: moss - Vinciguerra and De Francisci (1973)), **Bulgaria** (*Fagusorientalis* L., *Caprinusbetulus* L., with undergrowth of *Daphnepontica* L., *Cyclamencoum* Mill., *Asperulaodorata* L., 385 m a.s.l. - Iliev and Ilieva (2014)) and **Hungary** (moss - Andrássy (2009b)).

### Enchodelus ameliae

Guerrero, Liébanas & Peña-Santiago, 2008

03ED01D8-C065-538E-ACA6-434563FA51F3

6F323908-ABDA-444D-8A88-0FC39EA230E4

https://www.catalogueoflife.org/data/taxon/8GWTW

https://www.gbif.org/species/10880151

https://sibils.text-analytics.ch/search/?query=enchodelus%20ameliae#results-section

Enchodelus ameliae Guerrero, Liébanas & Peña-Santiago, 2008; Guerrero et al. (2008a) BLR: https://doi.org/10.5281/zenodo.8114884
Enchodelus ameliae ; *Peña-Santiago (2021b)*:206 BLR: https://doi.org/10.5281/zenodo.11191879

#### Distribution

This species is a part of Western Palearctic nematode fauna, reported only from its type localty in **Spain** (type habitat: hedgehog heath, 1950 m a.s.l. - Guerrero et al. (2008a)).

### Enchodelus analatus

(Ditlevsen, 1927) Thorne, 1939

38E94B85-0B85-50F6-93DC-C4BA35D3AD77

8C88711B-E0E5-4A2D-A8F8-32917C746512

https://www.catalogueoflife.org/data/taxon/9HDVL

https://www.gbif.org/species/2282678

https://sibils.text-analytics.ch/search/?query=enchodelus%20analatus#results-section

Dorylaimus (Doryllium) analatus Ditlevsen, 1927; Ditlevsen (1927) BLR: https://doi.org/10.5281/zenodo.10830636
Dorylaimellus analatus (Ditlevsen, 1927) Thorne & Swanger, 1936; Thorne and Swanger (1936) BLR: https://doi.org/10.5281/zenodo.10845799
Enchodelus (Rotundus) analatus (Ditlevsen, 1927) Thorne, 1939; Ahmad and Jairajpuri (1980) BLR: https://doi.org/10.5281/zenodo.8144936
Enchodelus analatus (Ditlevsen, 1927) Thorne, 1939; Thorne (1939) BLR: https://doi.org/10.5281/zenodo.11003139
Enchodelus analatus ; *Loof (1971)* BLR: https://doi.org/10.5281/zenodo.8152928
Enchodelus analatus ; *Eliava and Eliashvily (1990)*:51-52,96 BLR: https://doi.org/10.5281/zenodo.11003031
Enchodelus analatus ; *Holovachov (2014)*:22 [list] Plazi treatment
Enchodelus analatus ; Peña-Santiago (2021b):206 BLR: https://doi.org/10.5281/zenodo.11191883

#### Distribution

This species is a member of Nearctic nematode fauna: **Greenland** (type habitat: moss - Ditlevsen (1927)).

After its discovery, it was reported in Palearctic, mainly in the Arctic: **Norway** (**Spitzbergen**) (bare soil, sand, grasses (*Papaver*, *Silene*, *Saxifraga*, *Draba*, *Oxyria*, *Polygonum*), 20-350 m a.s.l. - Loof (1971)), **Romania** (beech - Popovici (1989), Popovici (1993)) and **Russia** (Arctic tundra - Kuzmin and Gagarin (1990)).

#### Taxon discussion

Originally described by Ditlevsen (1927) from Greenland, it was later reported from Spitzbergen (Loof 1971). Nevertheless, Guerrero et al. (2008a) raised doubt about the true identity of Loof’s material, which, in their opinion, was closer to *E.hopedorus*, this previously having been re-described by Guerrero and Peňa-Santiago (2007).

### Enchodelus arcticus

Nesterov, 1976

4EEB2D6F-CBD1-5EAF-A2B3-C084AA9D62FD

2BDEC04D-338C-4548-8E5F-BD7239F5DBCD

https://www.catalogueoflife.org/data/taxon/9J4KY

https://www.gbif.org/species/7018646

https://sibils.text-analytics.ch/search/?query=enchodelus%20arcticus#results-section

Enchodelus arcticus Nesterov, 1976; Nesterov (1976) BLR: https://doi.org/10.5281/zenodo.10849786
Enchodelus arcticus ; *Eliava and Eliashvily (1990)*:52,97 BLR: https://doi.org/10.5281/zenodo.11003031
Enchodelus arcticus ; *Peña-Santiago (2021b)*:206 BLR: https://doi.org/10.5281/zenodo.11192261

#### Distribution

*Enchodelusarcticus* is recorded from the Palearctic, more specifically from Arctic Russian territories and high latitudes: **Russia** (type habitat: soil under lichens (Polar Urals) and rhizosphere of herbaceous plants (Yamal Peninsula) - Nesterov (1976)).

#### Notes

This is the only described species that lacks male ventromedian supplements, but it should be noted that just one male specimen has been recorded.

### Enchodelus babakicus

Pedram, Niknam, Guerrero, Ye & Robbins, 2009

2B982903-5B97-5A96-8DE3-A851A06B88E8

EE731CB2-EB75-4800-B6C3-F3CD87686493

https://www.catalogueoflife.org/data/taxon/8GPPL

https://www.gbif.org/species/10704615

https://www.ebi.ac.uk/ena/browser/view/Taxon:592164

https://sibils.text-analytics.ch/search/?query=enchodelus+babakicus&tab=plazi#results-section

Enchodelus babakicus Pedram, Niknam, Guerrero, Ye & Robbins, 2009; Pedram et al. (2009) BLR: https://doi.org/10.5281/zenodo.8114779
Enchodelus babakicus ; Peña-Santiago (2021b):206 BLR: https://doi.org/10.5281/zenodo.11192263

#### Distribution

The species is a member of nematode fauna of Eastern Palearctic, found only in mountains of **Iran** (type habitat: rhizosphere of grasses from natural grasslands - Pedram et al. (2009)).

### Enchodelus brasiliensis

Meyl, 1957

D1CE3E45-574D-5C67-8D23-0B49A3E093B9

E425F950-4AD2-41AE-9483-F0C5192396B6

https://www.catalogueoflife.org/data/taxon/9J3NZ

https://www.gbif.org/species/7018530

Enchodelus brasiliensis Meyl, 1957; Meyl (1957) BLR:https://doi.org/10.5281/zenodo.10997965
Enchodelus brasiliensis ; Eliava and Eliashvily (1990):53,97 BLR: https://doi.org/10.5281/zenodo.11003031
Enchodelus brasiliensis ; Peña-Santiago (2021b):206 BLR: https://doi.org/10.5281/zenodo.11191887

#### Distribution

This is the only member of genus *Enchodelus* of Neotropical nematode fauna: **Brazil** (type habitat: wet sand - Meyl (1957)).

#### Taxon discussion

This species is the only representative of genus recorded from the Southern Hemisphere (Brazil), an interesting biogeographical singularity. Originally described on the basis of one female and three male specimens, Andrássy (1971) transferred it to *Rhyssocolpus*, but it better fits the *Enchodelus* diagnosis (very slender odontostyle, odontophore with flange-like extensions, short pharyngeal bulb, convex conoid tail).

### Enchodelus carpaticus

Ciobanu, Popovici, Guerrero & Peña-Santiago, 2010

4D4936D3-DE7D-50DD-B56B-8DC606D12DEF

8B206EBC-E0E8-433E-85E0-0A881D5B9483

https://www.catalogueoflife.org/data/taxon/9J4Q5

https://www.gbif.org/species/11820852

https://sibils.text-analytics.ch/search/?query=enchodelus%20carpaticus&tab=plazi#results-section

Enchodelus carpaticus Ciobanu, Popovici, Guerrero & Peña-Santiago, 2010; Ciobanu et al. (2010a) BLR: https://doi.org/10.5281/zenodo.8111703
Enchodelus carpaticus ; Peña-Santiago (2021b):206 BLR: https://doi.org/10.5281/zenodo.11192267

#### Distribution

This species is recorded in the Palearctic, with only one record from its type locality: **Romania** (type habitat: mountain grassland, 980 m a.s.l. - Ciobanu et al. (2010a)).

### Enchodelus decraemerae

Pourjam, Pedram, Vinciguerra & Robbins, 2010

419A9A0D-FA4F-5686-A4E7-563D3077520F

015B01B2-199B-48E8-819C-25D8C2F2AB2C

https://www.catalogueoflife.org/data/taxon/9J4HF

https://www.gbif.org/species/11690414

https://sibils.text-analytics.ch/search/?query=enchodelus%20decraemerae&tab=plazi#results-section

Enchodelus decraemerae Pourjam, Pedram, Vinciguerra & Robbins, 2010; Pourjam et al. (2010) BLR: https://doi.org/10.5281/zenodo.10716290
Enchodelus decraemerae ; Peña-Santiago (2021b):206 BLR: https://doi.org/10.5281/zenodo.11192271

#### Distribution

The species is a member of nematode fauna of West Palearctic: **Iran** (type habitat: rhizosphere of mosses on rocks - Pourjam et al. (2010)).

#### Taxon discussion

According to Pourjam et al. (2010), this species belongs to a group of species with very long odontostyle (61-67 μm) and conical tail, which Ahmad and Jairajpuri (1980) ascribed to the subgenusNepalus, now regarded identical with *Heterodorus*. Nevertheless, several remarkable traits (distinct flanges at odontophore base, tripartite uterus, 10-12 irregularly-spaced ventromedian supplements without hiatus) supports its inclusion in *Enchodelus*, although the conical tail with rounded tip is atypical in the genus. Thus, it is provisionally retained under *Enchodelus*, but further study based also on molecular data would elucidate its taxonomic status.

### Enchodelus distinctus

Ahmad & Jairajpuri, 1980

ADACFCF9-64F2-5F14-A606-2BE0D5FCC0F3

BF76039B-A151-458A-9041-A3D1B215B4E1

https://www.catalogueoflife.org/data/taxon/9J3YC

https://www.gbif.org/species/7018636

https://sibils.text-analytics.ch/search/?query=enchodelus%20distinctus&tab=plazi#results-section

Enchodelus (Enchodelus) distinctus Ahmad & Jairajpuri, 1980; Ahmad and Jairajpuri (1980) BLR: https://doi.org/10.5281/zenodo.8144946
Enchodelus distinctus ; Eliava and Eliashvily (1990):56,98 BLR: https://doi.org/10.5281/zenodo.11003031
Enchodelus distinctus ; Peña-Santiago (2021b):206 BLR: https://doi.org/10.5281/zenodo.11194433

#### Distribution

This species has been recorded only from Indomalayan region: **India** (type habitat: soil around roots of unidentified grasses, 4400 m a.s.l. - Ahmad and Jairajpuri (1980)).

#### Taxon discussion

This species is only known to occur in India and was described on the basis of a single female with bipartite uterus.

### Enchodelus georgensis

Eliava, Tskitishvili & Bagaturia, 2006

1AA40318-0BFE-5FA4-8098-ACB61034207A

EDE1423C-8D44-4CC7-A029-2D76F3DB7C55

https://www.catalogueoflife.org/data/taxon/9J4K6

https://www.gbif.org/species/7018645

Enchodelus georgensis Eliava, Tskitishvili & Bagaturia, 2006; Eliava et al. (2006) BLR: https://doi.org/10.5281/zenodo.11003168
Enchodelus georgensis ; *Peña-Santiago (2021b)*:207 BLR: https://doi.org/10.5281/zenodo.11193694

#### Distribution

*Enchodelusgeorgensis* is found only from the Palearctic, with a single report from **Georgia** (type habitat: deciduous forest, moss, firry forest, soil - Eliava et al. (2006)).

### Enchodelus groenlandicus

(Ditlevsen, 1927) Thorne, 1939

EAF845FD-107B-59CA-8C97-C73582ACF2C3

867FE891-B62E-4908-8943-4A7B3D922615

https://www.catalogueoflife.org/data/taxon/9J264

https://www.gbif.org/species/7018595

https://sibils.text-analytics.ch/search/?query=enchodelus%20groenlandicus&tab=plazi#results-section

Dorylaimus (Doryllium) groenlandicus Ditlevsen, 1927; Ditlevsen (1927) BLR: https://doi.org/10.5281/zenodo.10830630
Dorylaimellus groenlandicus (Ditlevsen, 1927) Thorne & Swanger, 1936; Thorne and Swanger (1936) BLR: https://doi.org/10.5281/zenodo.10845805
Enchodelus (Enchodelus) groenlandicus (Ditlevsen, 1927) Thorne, 1939
Enchodelus (Enchodelus) groenlandicus ; *Ahmad and Jairajpuri (1980)* BLR: https://doi.org/10.5281/zenodo.8144934
Enchodelus groenlandicus (Ditlevsen, 1927) Thorne, 1939; Thorne (1939) BLR: https://doi.org/10.5281/zenodo.11003133
Enchodelus groenlandicus ; *Eliava and Eliashvily (1990)*:57;98 BLR: https://doi.org/10.5281/zenodo.11003031
Enchodelus groenlandicus ; *Guerrero et al. (2008b)* BLR: https://doi.org/10.5281/zenodo.8111847
Enchodelus groenlandicus ; *Andrássy (2009a)* BLR: https://doi.org/10.5281/zenodo.10728535

Enchodelus groenlandicus ; *Pedram et al. (2011)*BLR: https://doi.org/10.5281/zenodo.8114666

Enchodelus groenlandicus ; *Elshishka et al. (2012)* Plazi treatment
Enchodelus groenlandicus ; *Holovachov (2014)*:22 [list] Plazi treatment
Enchodelus groenlandicus ; *Peña-Santiago (2021b)*:207 BLR: https://doi.org/10.5281/zenodo.11192354

#### Distribution

This species is representative of Holarctic nematode fauna, reported for the first time from **Greenland** (type habitat: a brook - Ditlevsen (1927)) and later from: **Albania** (soil from around beech trees, soil around brook, 950-1500 m a.s.l. - Andrássy (2009a)), **Iran** (the rhizosphere of grasses - Pedram et al. (2011)), **Russia** (Arctic tundra - Kuzmin and Gagarin (1990) / Arctic polygonal nival desert, associated with *Deschampsiaborealis* (Trautv.) Roshev., *Gymnomitriumcoraloides* Nees., *Cladonia* sp., 750 m a.s.l - Elshishka et al. (2012)), **Spain** (meadow and hedgehog heath, 955-2450 m a.s.l. - Guerrero et al. (2008b)) and **Uzbekistan** (a cotton field - Khakimov (1973)).

#### Taxon discussion

Originally described by Ditlevsen (1927), based on a single female specimen from Disko Island, Greenland, with Guerrero et al. (2008b) providing a more detailed description of Iberian populations. Later, the species was also reported, along with descriptions, from Albania, Iran and Russian Arctic (polygonal polar desert on Plateau Putorana) (Andrássy 2009a, Pedram et al. 2011, Elshishka et al. 2012, respectively), but Iranian specimens deviate in many morphometric characters from the type population and subsequent records. Besides, the species was recorded in a cotton field in Uzbekistan (Khakimov (1973)), but Guerrero et al. (2008b) suggested that this material belonged to a different species (characterised by a short body and post-equatorial vulva). Further, Andrássy (2009b) suggested that *E.groenlandicus* and *E.saxifragae* are identical.

#### Notes

Geographical distribution of *E.groenlandicus* shows a remarkable disjunction pattern as it occurs at high altitudes 950 m - 2450 m a.s.l. in southern Europe and Iran and at high latitudes in the Arctic Polar Region (Putorana Plateau and Greenland). Guerrero et al. (2008b) hypothesised that such distribution might stem from quaternary glacial events.

### Enchodelus hopedoroides

Altherr, 1963

2181C802-6DC7-5E25-8C5B-1E86EE4BBA09

4BD2425E-388C-41F1-93EF-D709B1B18CB0

https://www.catalogueoflife.org/data/taxon/9HMJZ

https://www.gbif.org/species/4554561

https://sibils.text-analytics.ch/search/?query=enchodelus%20hopedoroides&tab=plazi#results-section

Enchodelus (Enchodelus) hopedoroides Altherr, 1963 (Ahmad & Jairajpuri, 1980); Ahmad and Jairajpuri (1980) BLR: https://doi.org/10.5281/zenodo.8144934
Enchodelus hopedoroides Altherr, 1963; Altherr (1963) BLR: https://doi.org/10.5281/zenodo.8117912
Enchodelus hopedoroides ; Eliava and Eliashvily (1990):57,98 BLR: https://doi.org/10.5281/zenodo.11003031
Enchodelus hopedoroides ; *Guerrero et al. (2008a)* BLR: https://doi.org/10.5281/zenodo.8114882
Enchodelus hopedoroides ; Peña-Santiago (2021b):207 BLR: https://doi.org/10.5281/zenodo.11191895

#### Distribution

*Enchodelushopedoroides* is a part of Palearctic nematode fauna, reported only from Europe: **Switzerland** (type habitat: subalpine forest, *Calamagrostisvillosa* (Chaix) Gmel., grasses, 2170 m a.s.l. - Altherr (1963)) and **Spain** (wet meadow, 2800 m a.s.l. - Guerrero et al. (2008a)).

#### Taxon discussion

This species was originally described by Altherr (1963) in Switzerland. Later, Guerrero et al. (2008a) re-examined its type material and studied Iberian specimens.

### Enchodelus hopedorus

(Thorne, 1929) Thorne, 1939

D606BB56-865A-59D9-BEE0-6D64BFC6C658

BB74D958-FD13-49C9-9D2F-57B8DC1572C3

https://www.catalogueoflife.org/data/taxon/9HJ34

https://www.gbif.org/species/4554550

https://www.ebi.ac.uk/ena/browser/view/211319

https://sibils.text-analytics.ch/search/?query=enchodelus%20hopedorus&tab=plazi#results-section

Dorylaimellus hopedorus Thorne, 1929; Thorne (1929) BLR: https://doi.org/10.5281/zenodo.10797322
Enchodelus (Enchodelus) hopedorus (Thorne, 1929) Thorne, 1939
Enchodelus (Enchodelus) hopedorus ; *Ahmad and Jairajpuri (1980)* BLR: https://doi.org/10.5281/zenodo.8144934
Enchodelus (Enchodelus) hopedorus ; *Choi et al. (1997)* BLR: https://doi.org/10.5281/zenodo.10822468
Enchodelus hopedorus (Thorne, 1929) Thorne, 1939; Thorne (1939) BLR: https://doi.org/10.5281/zenodo.11003129
Enchodelus hopedorus ; *Brzeski (1963a)* BLR: https://doi.org/10.5281/zenodo.10869685
Enchodelus hopedorus ; *Zullini (1973)* BLR: https://doi.org/10.5281/zenodo.10845257
Enchodelus hopedorus ; Andrássy (1978):116 [list]
Enchodelus hopedorus ; *Winiszewska-Slipinska (1987)* BLR: https://doi.org/10.5281/zenodo.10854938
Enchodelus hopedorus ; Eliava and Eliashvily (1990):58,98 BLR: https://doi.org/10.5281/zenodo.11003031
Enchodelus hopedorus ; *Guerrero and Peňa-Santiago (2007)* BLR: https://doi.org/10.5281/zenodo.8111780
Enchodelus hopedorus ; Peña-Santiago (2021b):207 BLR: https://doi.org/10.5281/zenodo.11194191

#### Distribution

A member of Holarctic nematode fauna, reported from: **USA** (type habitat: roots of alpine plants and moss, 4344 m a.s.l. - Thorne (1929) / deciduous forests - Johnson et al. (1972)), **North America** (freshwater habitats - Esser and Buckingham (1987)), **Georgia** (Eliava and Eliashvily (1990)), **Korea** (*Pinusdensiflora* Siebold & Zucc. - Choi et al. (1997)) and **Poland** (*Sphagnum* spp. - Brzeski (1963a) / oak-hornbeam forest litter and peat bog litter - Winiszewska-Slipinska (1987)), as well as from the high Himalaya, which form part of the boundary between the Palearctic and Indomalayan Regions: **Nepal** (soil from *Rhododendron* spp., *Betulautilis* D. Don, *Abies* sp., 4100 m a.s.l. - Zullini (1973)).

#### Taxon discussion

After its original description in the USA, this species was recorded from different locations in Europe and Asia. Guerrero et al. (2008a) raised doubts regarding the identity of non-American material, with the possible exception of Polish material studied by Winiszewska-Slipinska (1987), which might belong to other species.

### Enchodelus laevis

Thorne, 1939

4989E810-BFFD-5A10-AC07-7423DC8CC295

E6295909-E23E-42DC-9F97-3E8A9F98B91D

https://www.catalogueoflife.org/data/taxon/8G859

https://www.gbif.org/species/7018633

https://sibils.text-analytics.ch/search/?query=enchodelus%20laevis&tab=plazi#results-section

Enchodelus (Rotundus) laevis Thorne, 1939 (Ahmad & Jairajpuri, 1980); Ahmad and Jairajpuri (1980) BLR: https://doi.org/10.5281/zenodo.8144936
Enchodelus knuppenburgensis Altherr in Altherr & Delamare-Deboutteville, 1972; Altherr and Delamare-Deboutteville (1972) BLR:https://doi.org/10.5281/zenodo.10723432
Enchodelus knuppenburgensis ; Eliava and Eliashvily (1990):59 BLR: https://doi.org/10.5281/zenodo.11003031
Enchodelus laevis Thorne, 1939; Thorne (1939) BLR: https://doi.org/10.5281/zenodo.11003143
Enchodelus laevis ; Eliava and Eliashvily (1990):59,99 BLR: https://doi.org/10.5281/zenodo.11003031
Enchodelus laevis ; *Brzeski (1992)* BLR: https://doi.org/10.5281/zenodo.8122483
Enchodelus laevis ; Peña-Santiago (2021b):207 BLR: https://doi.org/10.5281/zenodo.11194193

#### Distribution

Originally recovered from Nearctic: **USA** (type habitat: fresh water stream bank - Thorne (1939) / Altherr and Delamare-Deboutteville (1972)); subsequently, this species is also recorded in Eastern Palearctic: **Korea** (moss - Brzeski (1992)).

#### Taxon discussion

This species was briefly described from the USA by Thorne (1939) on the basis of one female specimen. After that, Altherr, in Altherr and Delamare-Deboutteville (1972), studied one male from the USA too that was described as *E.knuppenburgensis*, very close to *E.laevis* in its general morphology. Thus, Altherr suggested that his male might be conspecific with the *E.laevis* female. Brzeski (1992) described five females and one male specimens of *E.laevis* from Korea and regarded both species as identical.

### Enchodelus longispiculus

Guerrero, Liébanas & Peña-Santiago, 2008

82D5D1BD-1333-5960-8090-70A89111825E

F7B216E2-08F1-4A55-AA31-0F838A47207B

https://www.catalogueoflife.org/data/taxon/8GPPM

https://www.gbif.org/species/10810810

https://www.ebi.ac.uk/ena/browser/view/Taxon:1068861

https://sibils.text-analytics.ch/search/?query=enchodelus%20longispiculus&tab=plazi#results-section

Enchodelus longispiculus Guerrero, Liébanas & Peña-Santiago, 2008; Guerrero et al. (2008a) BLR: https://doi.org/10.5281/zenodo.8114886
Enchodelus longispiculus ; *Ciobanu et al. (2010b)* BLR: https://doi.org/10.5281/zenodo.8111641
Enchodelus longispiculus ; *Pedram et al. (2011)* BLR: https://doi.org/10.5281/zenodo.8114668
Enchodelus longispiculus ; *Peña-Santiago (2021b)*:207-208 BLR: https://doi.org/10.5281/zenodo.11191897

#### Distribution

A typical member of Palearctic nematode fauna: **Spain** (type habitat: wet meadow, 2925 m a.s.l., other microhabitats: gorse scrubland and wet meadows, 2590-2700 m a.s.l. - Guerrero et al. (2008a)), **Iran** (rhizosphere of grasses - Pedram et al. (2011)) and **Romania** (beech forest, cliff vegetation, grassland, 400-2000 m a.s.l. - Ciobanu et al. (2010b)).

#### Taxon discussion

Originally described from Spain, this species was later recorded in Romania and Iran. Pedram et al. (2011) provided its first molecular characterisation, based on the 18S rDNA, ITS and partial 5.8S gene.

### Enchodelus lucinensis

Popovici, 1978

5FC82370-10D7-5984-B78F-8A5537EE66DE

573AEEF2-A7E8-4E73-82E8-D24E34F897ED

https://www.catalogueoflife.org/data/taxon/9HMK3

https://www.gbif.org/species/4554528

https://sibils.text-analytics.ch/search/?query=enchodelus%20lucinensis%C2%A0&tab=plazi#results-section

Enchodelus lucinensis Popovici, 1978; Popovici (1978) BLR: https://doi.org/10.5281/zenodo.8122645
Enchodelus lucinensis ; Eliava and Eliashvily (1990):60,99 BLR: https://doi.org/10.5281/zenodo.11003031
Enchodelus lucinensis ; Ciobanu et al. (2010b) BLR: https://doi.org/10.5281/zenodo.8111643
Enchodelus lucinensis ; Peña-Santiago (2021b):208 BLR: https://doi.org/10.5281/zenodo.11191899

#### Distribution

*Enchodeluslucinensis* is a member of Palearctic nematode fauna, found in different localities and habitats from: **Romania** (type habitat: peat bog, other habitat: mezohygrophilous meadow*, 1050-1250 m a.s.l.* - Popovici (1978), Ciobanu et al. (2010b)*), **Slovakia** (alpine meadows, 1994-2200 m a.s.l. - Háněl (2017)) and **Turkey** (wildflower meadows, mountain grasslands, riverbed, 2500-4000 m a.s.l. - Çakmak et al. (2021)).

#### Taxon discussion

Originally described by Popovici (1978), the type material of this species was later re-examined by Ciobanu et al. (2010b). These authors considered that its separation from *E.teres* (due to brief original description) is very problematic and based on minor differences.

### Enchodelus makarovae

Elshishka, Lazarova & Peneva, 2012

8D5CC2C5-0E19-5B76-90D7-4E4BC12E7355

FFC630CE-A71F-4361-A611-8CA1862AE381

https://www.catalogueoflife.org/data/taxon/9HNCS

https://www.gbif.org/species/8848341

https://sibils.text-analytics.ch/search/?query=enchodelus+makarovae&tab=plazi#results-section

Enchodelus makarovae Elshishka, Lazarova & Peneva, 2012; Elshishka et al. (2012) Plazi treatment
Enchodelus makarovae ; Holovachov (2014):22 [list] Plazi treatment
Enchodelus makarovae ; Peña-Santiago (2021b):210 BLR: https://doi.org/10.5281/zenodo.11192362

#### Distribution

This species is a part of Palearctic nematode fauna, found only in an Arctic polar desert: **Russia** (Arctic, Severnaya Zemlya Archipelago, type habitat: polygonal polar desert with *Alopecurusalpinus* Sm., *G.coraloides*, *Lopadium* sp., *D.borealis*, black crust - Elshishka et al. (2012)).

### Enchodelus microdoroides

Baqri & Jairajpuri, 1974

FDAB2891-072F-5C2D-A6CF-0BA47D04010E

225D0BC0-58FF-45EB-A48D-243C1780F4AC

https://www.catalogueoflife.org/data/taxon/9J4QR

https://www.gbif.org/species/7018612

https://sibils.text-analytics.ch/search/?query=enchodelus+microdoroides&tab=plazi#results-section

Enchodelus (Enchodelus) microdoroides Baqri & Jairajpuri, 1974 (Ahmad & Jairajpuri, 1980); Ahmad and Jairajpuri (1980) BLR: https://doi.org/10.5281/zenodo.8144944
Enchodelus microdoroides Baqri & Jairajpuri, 1974; Baqri and Jairajpuri (1974) BLR: https://doi.org/10.5281/zenodo.8117932
Enchodelus microdoroides ; Eliava and Eliashvily (1990):62,100 BLR: https://doi.org/10.5281/zenodo.11003031
Enchodelus microdoroides ; Eliava and Eliashvily (1990)Brzeski (1992) BLR: https://doi.org/10.5281/zenodo.8122485
Enchodelus microdoroides ; Eliava and Eliashvily (1990)Peña-Santiago (2021b):210 BLR: https://doi.org/10.5281/zenodo.11198490

#### Distribution

*Enchodelusmicrodoroides* is a representative of Indomalayan nematode fauna: **India** (type habitat: soil around roots of barley, 549 m a.s.l., other habitat: grasses, 2100 m a.s.l. - Baqri and Jairajpuri (1974) / grasses and mosses - Ahmad and Jairajpuri (1980)); however, there is a single report from the Eastern Palearctic: **Korea** (moss - Brzeski (1992)).

### Enchodelus parahopedoroides

Ciobanu, Popovici, Guerrero & Peña-Santiago, 2010

0650CEA0-D896-5D72-8B79-79785F725CDE

14062C06-C50A-417C-94B2-5824024030A6

https://www.catalogueoflife.org/data/taxon/9J4BK

https://www.gbif.org/species/11798772

https://sibils.text-analytics.ch/search/?query=enchodelus%20parahopedoroides&tab=plazi#results-section

Enchodelus parahopedoroides Ciobanu, Popovici, Guerrero & Peña-Santiago, 2010; Ciobanu et al. (2010b) BLR: https://doi.org/10.5281/zenodo.8111645
Enchodelus parahopedoroides ; *Peña-Santiago (2021b)*:211 BLR:  https://doi.org/10.5281/zenodo.11191909

#### Distribution

This species is reported from West Palearctic Region: **Romania** (type habitat: cliff vegetation, 2200 m a.s.l., other habitats: spruce forest, mountain grassland, 1000-2000 m a.s.l. - Ciobanu et al. (2010b)).

### Enchodelus parateres

Baqri & Jairajpuri, 1974

0E049F23-A87E-5973-AC6C-21C9673C78D1

5E901204-F5D0-44EB-86CC-57850518D76E

https://www.catalogueoflife.org/data/taxon/9J3QS

https://www.gbif.org/species/7018643

https://sibils.text-analytics.ch/search/?query=enchodelus+parateres&tab=plazi#results-section

Enchodelus (Rotundus) parateres Baqri & Jairajpuri, 1974 (Ahmad & Jairajpuri, 1980); Ahmad and Jairajpuri (1980) Plazi treatment
Enchodelus parateres Baqri & Jairajpuri, 1974; Baqri and Jairajpuri (1974) BLR: https://doi.org/10.5281/zenodo.8117930
Enchodelus parateres ; Eliava and Eliashvily (1990):64,100 BLR: https://doi.org/10.5281/zenodo.11003031
Enchodelus parateres ; *Jairajpuri and Ahmad (1992)*:182 [figure] BLR: https://doi.org/10.5281/zenodo.10850166
Enchodelus parateres ; *Holovachov (2014)*:22 [list] Plazi treatment
Enchodelus parateres ; Peña-Santiago (2021b):211 BLR: https://doi.org/10.5281/zenodo.11191913

#### Distribution

*Enchodelusparateres* is a member of Indomalayan nematode fauna: **India** (type habitat: soil around roots of weeds and mosses, 2600 m a.s.l. - Baqri and Jairajpuri (1974) / soil around the roots of wild fruit trees, 1415 m a.s.l. - Ahmad and Jairajpuri (1980)).

The species was also recorded in Palearctic: **Romania** (grassland, mix forest, 1050 m a.s.l. - Ciobanu and Popovici (2017)) and **Russia** (Arctic tundra - Kuzmin (1986), Kuzmin and Gagarin (1990)).

### Enchodelus parvus

Loof, 1971

27BB1497-0CB8-5DCA-9294-2B83736E7BA0

66A06F84-3034-47C8-828C-880B6885AF3F

https://www.catalogueoflife.org/data/taxon/8GNN3

https://www.gbif.org/species/4554531

https://sibils.text-analytics.ch/search/?query=enchodelus%20parvus#results-section

Enchodelus (Rotundus) parvus Loof, 1971 (Ahmad & Jairajpuri, 1980); Ahmad and Jairajpuri (1980) BLR: https://doi.org/10.5281/zenodo.8144936
Enchodelus parvus Loof, 1971; Loof (1971) BLR: https://doi.org/10.5281/zenodo.8152930
Enchodelus parvus ; Eliava and Eliashvily (1990):65,101 BLR: https://doi.org/10.5281/zenodo.11003031
Enchodelus parvus ; *Holovachov (2014)*:22 [list] Plazi treatment
Enchodelus parvus ; Peña-Santiago (2021b):211 BLR: https://doi.org/10.5281/zenodo.11191917

#### Distribution

This species was reported in Palearctic, mainly in Arctic tundra: **Norway** (**Spitzbergen**) (type habitat: bare soil, 150 m a.s.l., other habitats: grass tufts and mosses, *Saxifraga* sp. - Loof (1971)), **Russia** (Arctic tundra - Kuzmin (1986), Kuzmin and Gagarin (1990)) and **Uzbekistan** (cotton - Mavlyanov et al. 1989). The last record requires confirmation.

### Enchodelus ponorensis

Popovici, 1995

39C30753-F945-5345-B6FF-2D1FACD085AC

E1EC7E6D-6D62-46AA-888B-65E7CDD3BCD5

https://www.catalogueoflife.org/data/taxon/9HMK6

https://www.gbif.org/species/4554555

https://sibils.text-analytics.ch/search/?query=enchodelus%20ponorensis#results-section

Enchodelus ponorensis Popovici, 1995; Popovici (1995a) BLR: https://doi.org/10.5281/zenodo.8125533
Enchodelus ponorensis ; *Ciobanu et al. (2010b)* BLR: https://doi.org/10.5281/zenodo.8111647
Enchodelus ponorensis ; Peña-Santiago (2021b):211 BLR: https://doi.org/10.5281/zenodo.11191919

#### Distribution

This species is reported from Western Palearctic Region: **Romania** (type habitat: mountain grassland, 1000 m a.s.l. - Popovici (1995a)).

#### Taxon discussion

Ciobanu et al. (2010b) re-examined type material of this species only known to occur in Romania. It is an atypical representative of the genus due to its short bipartite uterus, long pharyngeal expansion (ca. two-fifths of pharynx length) and longer and conoid tail, but other relevant traits (lip region shape, double guiding ring, flanged odontophore etc.) fit well the *Enchodelus* diagnosis.

### Enchodelus sardashtensis

Pedram, Pourjam, Robbins, Ye & Peña-Santiago, 2011

F7C6B8D2-6704-5CC9-9D06-6CF6E0CECEA6

89A41CD5-08E3-4C72-A003-C205D15FD8B9

https://www.catalogueoflife.org/data/taxon/8GPPN

https://www.gbif.org/species/10787556

https://www.ebi.ac.uk/ena/browser/view/Taxon:1068862

https://sibils.text-analytics.ch/search/?query=enchodelus%20sardashtensis%20&tab=plazi#results-section

Enchodelus sardashtensis Pedram, Pourjam, Robbins, Ye & Peña-Santiago, 2011; Pedram et al. (2011) BLR:https://doi.org/10.5281/zenodo.8114670
Enchodelus sardashtensis ; Peña-Santiago (2021b):211 BLR: https://doi.org/10.5281/zenodo.11194197

#### Distribution

This species is reported from Eastern Palearctic, only known from its type locality: **Iran** (type habitat: rhizosphere soil of grasses - Pedram et al. (2011)).

### Enchodelus saxifragae

Popovici, 1995

736F85E8-0CFD-53E6-BBEF-4B25F269E3DF

8F559D50-234C-48A9-B99A-D49E70C99EAB

https://www.catalogueoflife.org/data/taxon/9HJ35

https://www.gbif.org/species/4554544

https://www.ebi.ac.uk/ena/browser/view/Taxon:1440451

https://sibils.text-analytics.ch/search/?query=enchodelus%20saxifragae#results-section

Enchodelus saxifragae Popovici, 1995; Popovici (1995a) BLR: https://doi.org/10.5281/zenodo.8125535
Enchodelus saxifragae ; Guerrero et al. (2008b) BLR: https://doi.org/10.5281/zenodo.811185
Enchodelus saxifragae ; Ciobanu et al. (2010a) BLR: https://doi.org/10.5281/zenodo.8111707
Enchodelus saxifragae ; Peña-Santiago et al. (2015):147 [figure] BLR:https://doi.org/10.5281/zenodo.10698092
Enchodelus saxifragae ; Peña-Santiago (2021b):211 BLR: https://doi.org/10.5281/zenodo.11191923

#### Distribution

*Enchodelussaxifragae* is distributed in Western Palearctic, reported from different localities and habitats: **Romania** (type habitat: subalpine grassland on limestone, under *Saxifragamoschata* Wulf., 1950-2000 m a.s.l. - Popovici (1995a), Popovici and Ciobanu (1997) / grasslands - Popovici (1998), Ciobanu and Popovici (2001) / cliff vegetation, subalpine meadow, 1450-2000 m a.s.l. - Ciobanu et al. (2010a)) and **Spain** (hedgehog heath, psychroxerophilous pasture, pine forest with savin juniper, 1550-3350 m a.s.l. - Guerrero et al. (2008b), Peña-Santiago et al. (2015)).

#### Taxon discussion

This species, originally described and later repeatedly recorded in Romania, is also known to occur in Spain. Ciobanu et al. (2010a) re-examined its type material and noted the peculiar shape of the lateral guiding pieces, which was regarded as a relevant diagnostic feature. It is very close to *E.groenlandicus*, but it differs from this in lip region shape, degree of sclerotisations of the *pars refringens vaginae* and the presence of males. Peña-Santiago et al. (2015) provided its first molecular (18S rDNA) study.

### Enchodelus teres

Thorne, 1939

3768DFE9-F7AF-57E0-AF8D-7867D3CD93DE

8711F9F6-2790-4774-B3CD-8865203DBAD3

https://www.catalogueoflife.org/data/taxon/9HMK7

https://www.gbif.org/species/4554532

https://sibils.text-analytics.ch/search/?query=enchodelus%20teres#results-section

Enchodelus (Rotundus) teres Thorne, 1939 (Ahmad & Jairajpuri, 1980); Ahmad and Jairajpuri (1980) BLR: https://doi.org/10.5281/zenodo.8144936
Enchodelus teres Thorne, 1939; Thorne (1939) BLR: https://doi.org/10.5281/zenodo.11003135
Enchodelus teres ; Zullini (1970) BLR: https://doi.org/10.5281/zenodo.10850514
Enchodelus teres ; Eliava and Eliashvily (1990):67,101 BLR: https://doi.org/10.5281/zenodo.11003031
Enchodelus teres ; *Holovachov (2014)*:23 [list] Plazi treatment
Enchodelus teres ; Peña-Santiago (2021b):211 BLR: https://doi.org/10.5281/zenodo.11194199

#### Distribution

Holarctic species. First recovered in Nearctic: **USA** (type habitat: soil around root of alpine plants, 3440 m a.s.l. - Thorne (1939) / *Ambrosiapsilostachya* DC. - Orr and Dickerson (1967)) and also reported from few localities of Palearctic: **Italy** (moss, 1340 m a.s.l. - Zullini (1970)) and **Russia** (Arctic tundra - Kuzmin (1973), Kuzmin and Gagarin (1990)).

#### Taxon discussion

Available information about this species is very limited. On the one hand, the original description of American specimens lacks many details. On the other hand, its later records only provided some Demanian indices, if anything.

### Enchodelus vestibulifer

Altherr, 1952

2C368541-DF05-58D7-928F-CE883C14D6DD

7376F631-5490-452C-A770-2268F6245BEF

https://www.catalogueoflife.org/data/taxon/9HMK9

https://www.gbif.org/species/4554545

https://sibils.text-analytics.ch/search/?query=Enchodelus%20vestibulifer#results-section

Enchodelus vestibulifer Altherr, 1952; Altherr (1952) BLR: https://doi.org/10.5281/zenodo.10813922
Enchodelus vestibulifer ; Meyl (1961) BLR: https://doi.org/10.5281/zenodo.11015551
Enchodelus vestibulifer ; Eliava and Eliashvily (1990):68,102 BLR: https://doi.org/10.5281/zenodo.11003031
Enchodelus vestibulifer ; Peña-Santiago (2021b):211

#### Description

Material examined. One female in poor condition, mounted on one slide labelled *Enchodelusvestibulifer* n. sp., and collected from Parc National, st. 39 in July 1948 (Fig. 1).

**Figure 5.**
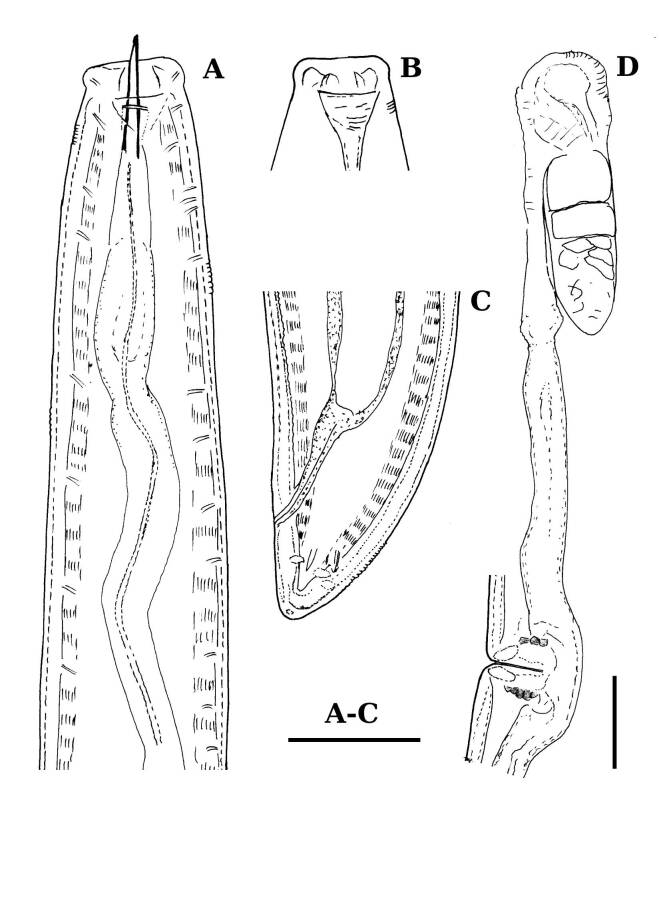
*Enchodelusvestibulifer* Altherr, 1952, female. **A** Anterior region; **B** Amphid; **C** Tail end; **D** Anterior genital branch. Scale bars: **A-C** 25 µm; **D** 25 µm.

Measurements: L = 1.36 mm, a = 32.4, b = 3.8, c = 61.8, c’ = 1.0, V = 53%, neck length = 355 µm (Fig. 5).

**Figure 6.**
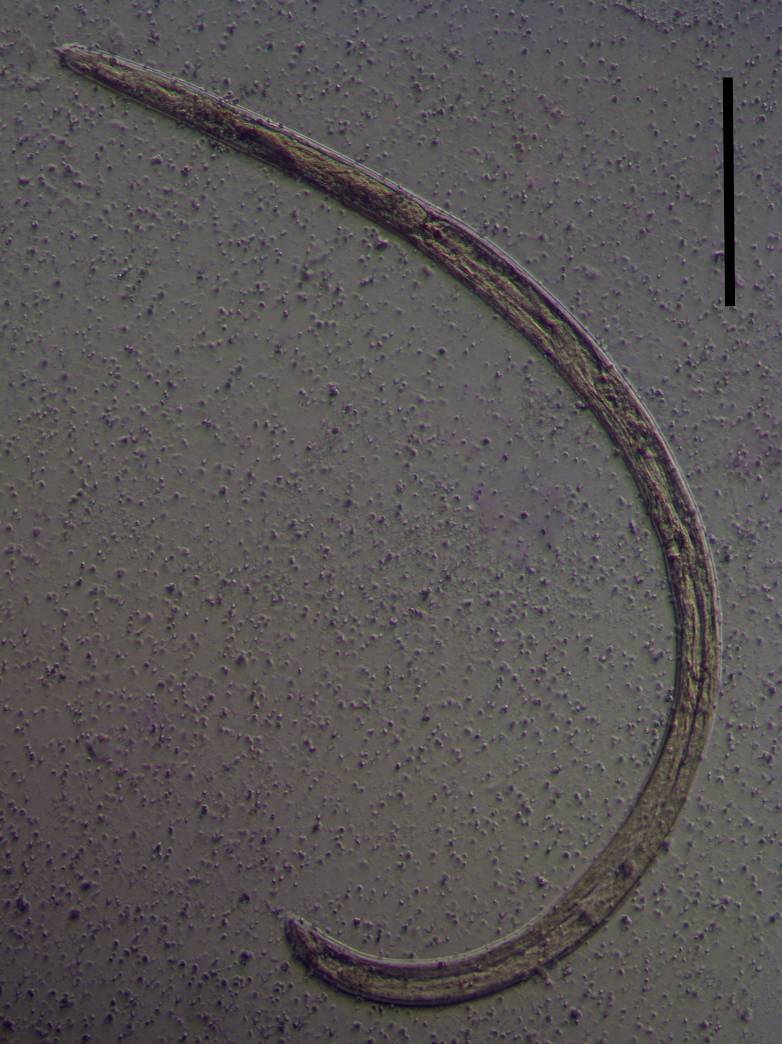
*Enchodelusvestibulifer* Altherr, 1952, entire body. Scale bar: 200 µm.

**Figure 7.**
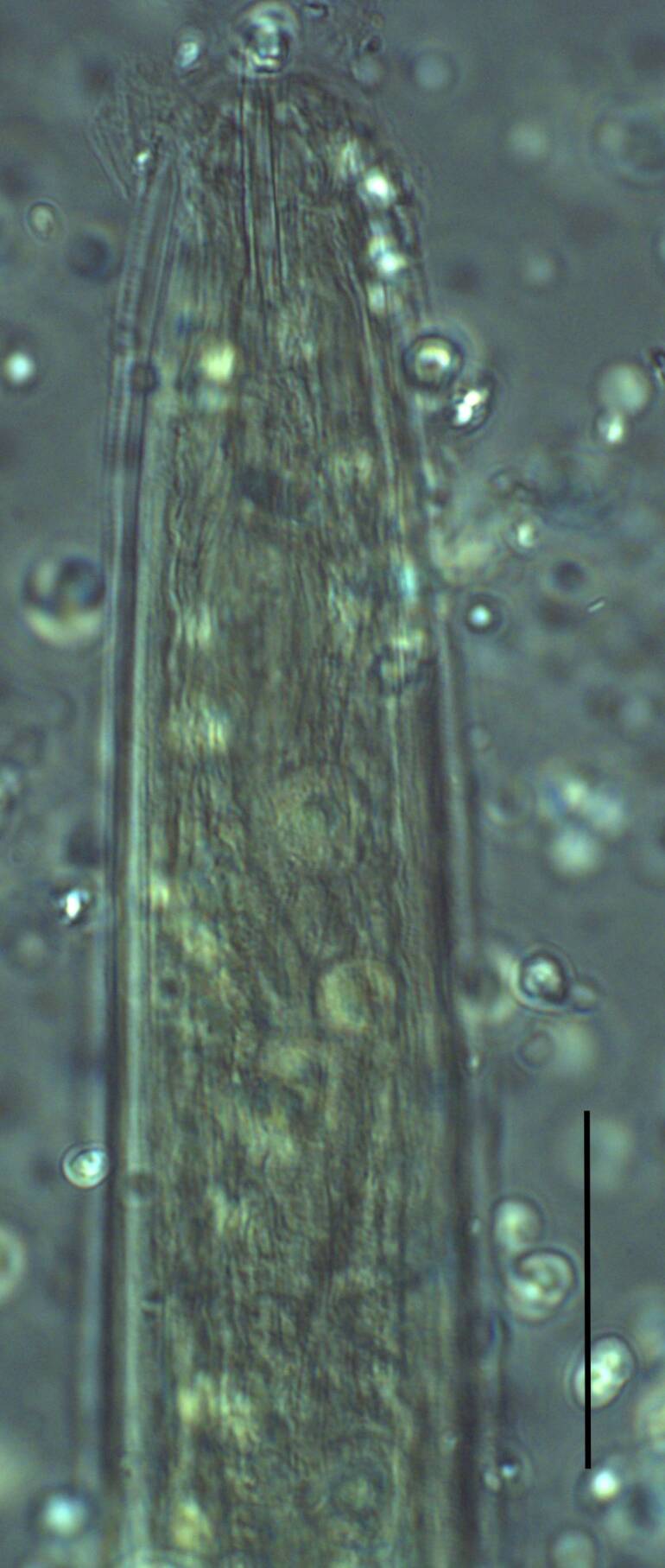
*Enchodelusvestibulifer* Altherr, 1952, anterior end. Scale bar: 30 µm.

**Figure 8.**
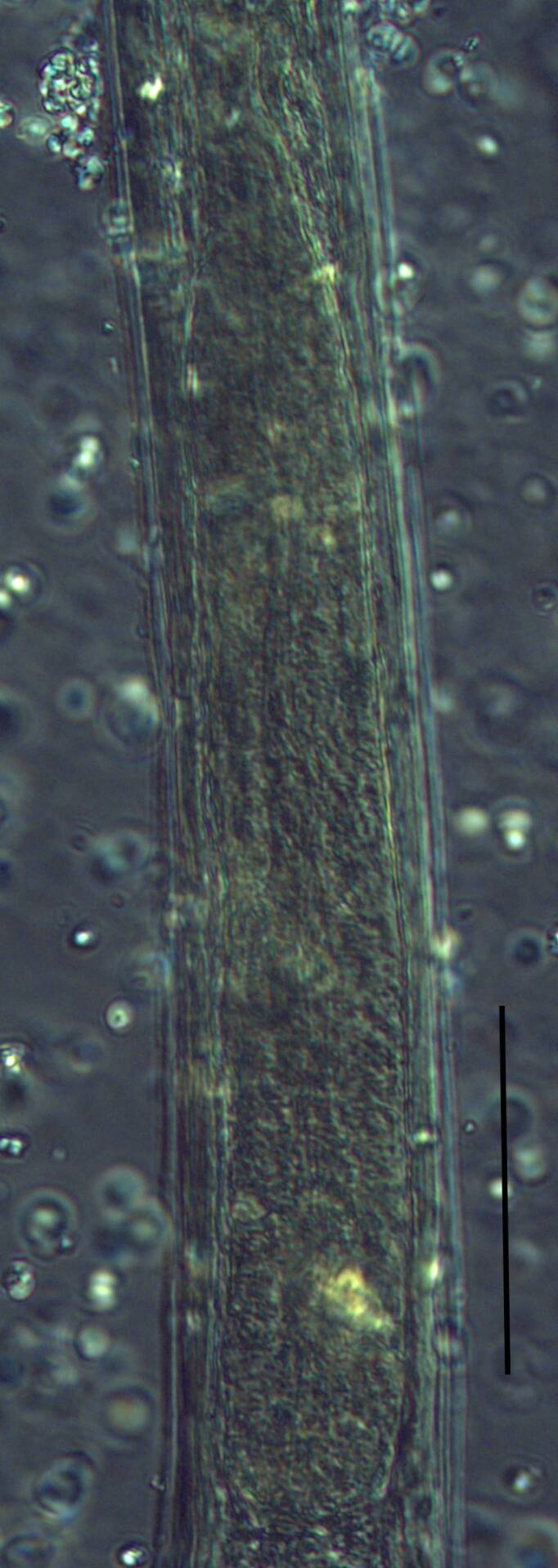
*Enchodelusvestibulifer* Altherr, 1952, pharyngeal expansion. Scale bar: 50 µm.

**Figure 9.**
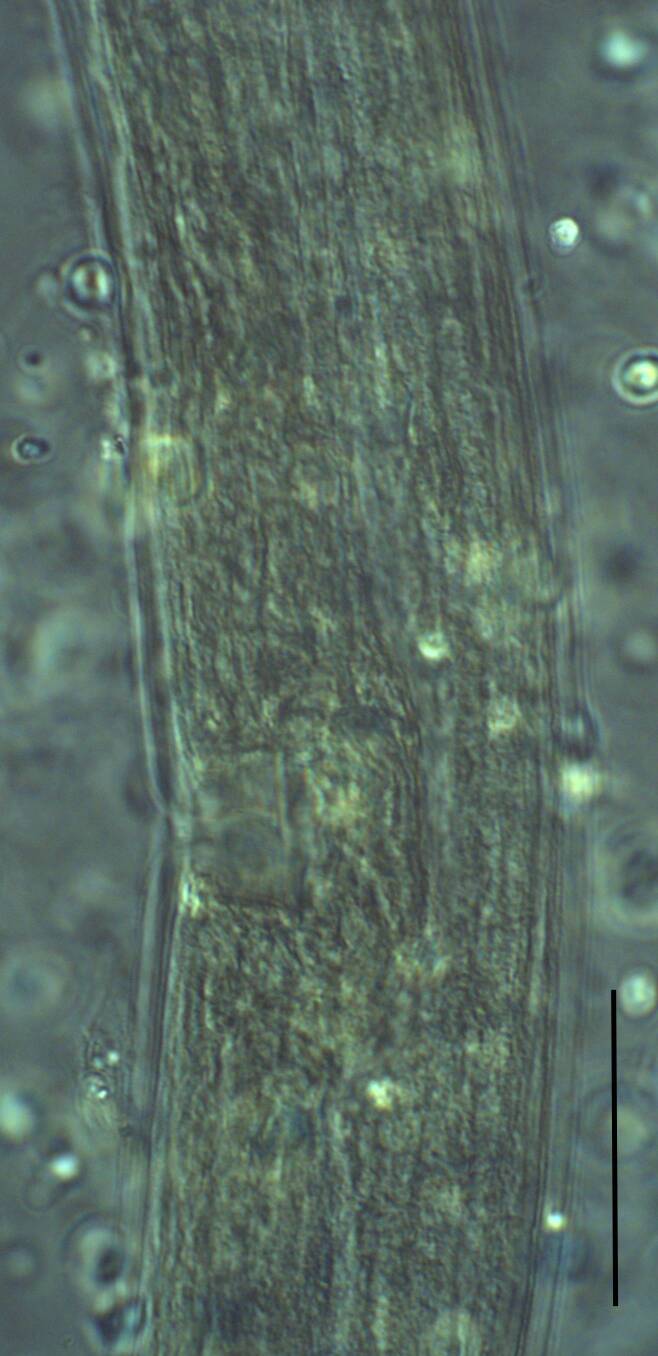
*Enchodelusvestibulifer* Altherr, 1952, vulval region. Scale bar: 30 µm.

**Figure 10.**
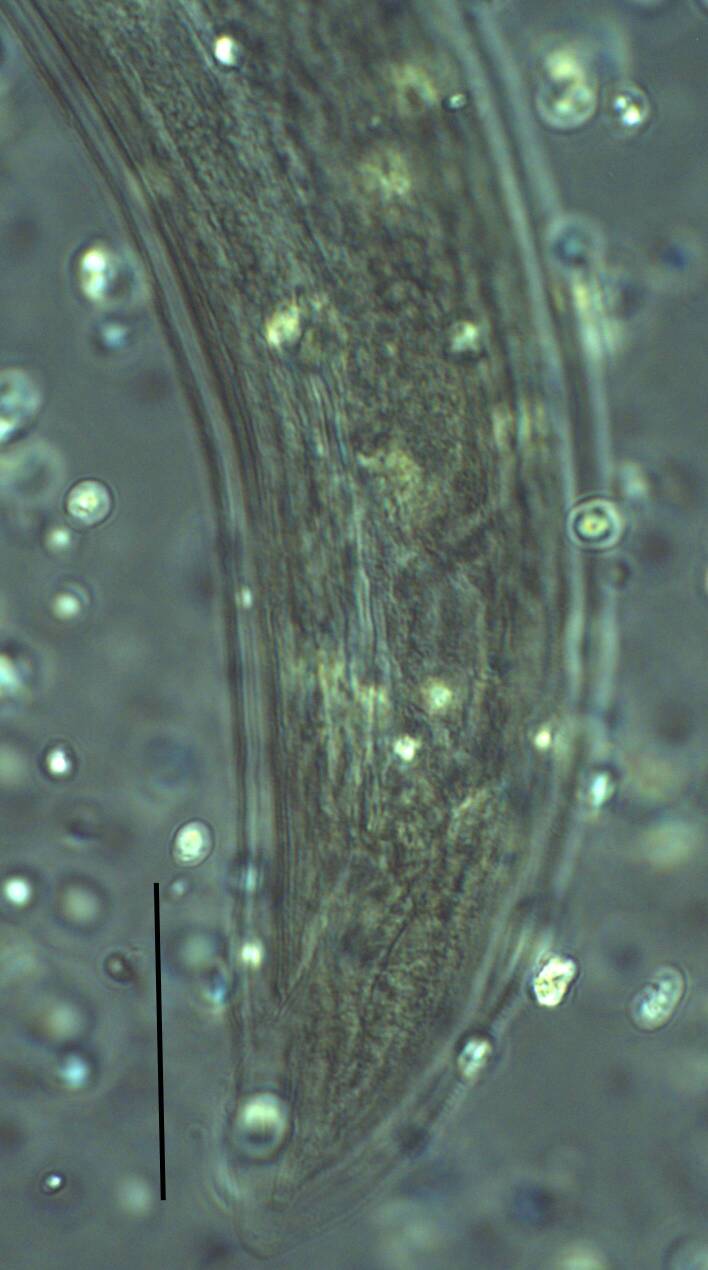
*Enchodelusvestibulifer* Altherr, 1952, posterior end. Scale bar: 30 µm.

Habitus curved ventrally after fixation, adopting an open C-shape (Fig. 6). Cuticle consisting of two layers, its outer layer provided with distinct transverse striations. Lip region 16 µm wide, offset by a shallow depression (Fig. 7). Cheilostom a wide cavity. Amphidial fovea funnel shape. Odontostyle 24 µm long, strong with wide lumen, straight, 1.5 times lip region diameter, 1.8% of body length. Guiding ring double. Anterior region of pharynx enlarging gradually; pharyngeal basal expansion 174 µm, occupying about half of total neck length, bearing muscular sheath (Fig. 8). Pharyngeal gland nuclei not visible, their orifices obscure. Cardia rounded. A disc-like structure separating the pharyngeal base from cardia present. Female genital system diovarian. Uteri not differentiated. Vagina extending inwards for 62% of body diameter, *pars refringens vaginae* not well preserved (Fig. 9). Prerectum 1.4, rectum 1.3 times anal body diameter long. Tail conical with rounded tip, 22 µm long, 1.8% of body length (Fig. 10).

#### Distribution

This species is a part of Palearctic nematode fauna, currently known only from **Switzerland** (type habitat: soil of grasses; 1900 m a.s.l. - Altherr (1952)).

#### Taxon discussion

Altherr (1952) described this species on the basis of a single female from Switzerland and no later record exists. The female forms part of Altherr’s collection, deposited in the Museo Cantonale di Storia Naturale di Lugano (Switzerland). Its state of preservation is not good, so that some morphological features are difficult to appreciate with accuracy.

The re-examination of this material has provided some differences from Altherr's original description - shorter body length (L = 1.36 vs. 1.55 mm), values of de Man indices "c" are lower, ''a'', c', V are higher, size of vagina, saccate bodies described and illustrated in the original description are hardly visible, most likely due to flattening of the specimen and conservation.

Meyl (1961) considered this species as *species inquirenda*. New observations reveal that several relevant traits (strong odontostyle with wide lumen, comparatively long pharyngeal expansion occupying ca. one-half of the total neck length, tail conical with rounded tip) are not compatible with those characterising the genus *Enchodelus*. Thus, this species is herein regarded as *incertae sedis*.

### Enchodelus vesuvianus

(Cobb, 1893) Thorne, 1939

A922E21E-6638-570C-9A73-9B5AA4B915FE

BA5F7F84-F01C-4874-AE74-0E352B5A2A3A

https://www.catalogueoflife.org/data/taxon/9HMKB

https://www.gbif.org/species/4554533

https://sibils.text-analytics.ch/search/?query=enchodelus%20vesuvianus#results-section

Dorylaimus vesuvianus Cobb, 1893; Cobb (1893) BLR: https://doi.org/10.5281/zenodo.10853995
Dorylaimus vesuvianus ; *Steiner (1914)*:262 [list] BLR: https://doi.org/10.5281/zenodo.11003080
Dorylaimellus vesuvianus (Cobb, 1893) Thorne & Swanger, 1936; Thorne and Swanger (1936) BLR: https://doi.org/10.5281/zenodo.10845811
Enchodelus (Enchodelus) vesuvianus (Cobb, 1893) Thorne, 1939; Ahmad and Jairajpuri (1980) BLR: https://doi.org/10.5281/zenodo.8144934
Enchodelus vesuvianus (Cobb, 1893) Thorne, 1939; Thorne (1939) BLR: https://doi.org/10.5281/zenodo.11003126
Enchodelus vesuvianus ; *Meyl (1954)* BLR: https://doi.org/10.5281/zenodo.10928057
Enchodelus vesuvianus ; Meyl (1961) BLR: https://doi.org/10.5281/zenodo.11015551
Enchodelus vesuvianus ; Zullini (1970) BLR: https://doi.org/10.5281/zenodo.10850516
Enchodelus vesuvianus ; Vinciguerra (1972) BRL: https://doi.org/10.5281/zenodo.11028268
Enchodelus vesuvianus ; Vinciguerra and De Francisci (1973) BLR: https://doi.org/10.5281/zenodo.8143851
Enchodelus vesuvianus ; Zullini (1978) BLR: https://doi.org/10.5281/zenodo.10814618
Enchodelus vesuvianus ; Winiszewska-Slipinska (1987) BLR: https://doi.org/10.5281/zenodo.10854942
Enchodelus vesuvianus ; Eliava and Eliashvily (1990):69,102 BLR: https://doi.org/10.5281/zenodo.11003031
Enchodelus vesuvianus ; Andrássy (2009b) BLR: https://doi.org/10.5281/zenodo.10821951
Enchodelus vesuvianus ; Peña-Santiago (2021b):211-212 BLR: https://doi.org/10.5281/zenodo.11191929

#### Distribution

The species is widespread in southern and central parts of Western Palearctic: **Italy** (type habitat: moss - Cobb (1893) / moss - Meyl (1954), moss - Zullini (1970), Zullini (1971), Vinciguerra (1972) / moss - Vinciguerra and De Francisci (1973) / moss - Zullini (1978) / moss on rock - Barbuto and Zullini (2006) / alpine springs - sediments, mosses - Zullini et al. (2011)), **Hungary** (Andrássy (1973), Andrássy (2009b)), **Poland** (*Festucetaliavalesiacae* Soó, *Arrhenatherum* sp. - Winiszewska-Slipinska (1987)) and **Switzerland** (Steiner (1914)).

In Nearctic, it is reported from **the USA** (*Schizachyriumscoparium* (Michx.) Nash, *Symphoricarposorbiculatus* Moench - Orr and Dickerson (1967)).

#### Notes

*Enchodelusvesuvianus* was found and described for the first time from Mount Vesuvius, Italy. This species is widespread in Europe, mainly associated with mosses. It has also been reported from the USA, but without morphological and morphometric data.

### Enchodelus enguriensis

Elshishka, Mladenov, Altash, Tskitishvili, Álvarez-Ortega, Peña-Santiago & Peneva sp. nov.

1AAB3096-7E11-5918-9DB9-66EAA829D89B

92281C43-6B4F-47D0-B1DE-B957B7439CFF

#### Description

Material examined. Four females, five males and twelve juveniles (J1-J4) from Georgia.

Measurements (see Table 1)

**Figure 11.**
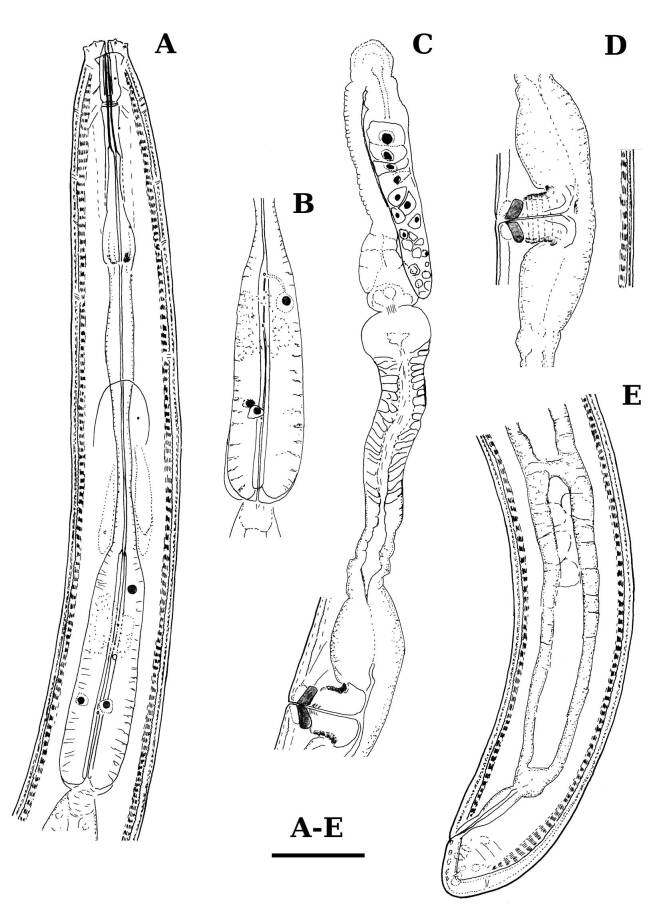
*Enchodelusenguriensis* sp. nov., female (Holotype). **A** Anterior region; **B** Pharyngeal expansion; **C** Anterior genital branch; **D** Vulval region; **E** Tail end. Scale bar: **A-E** 25 µm.

**Figure 12.**
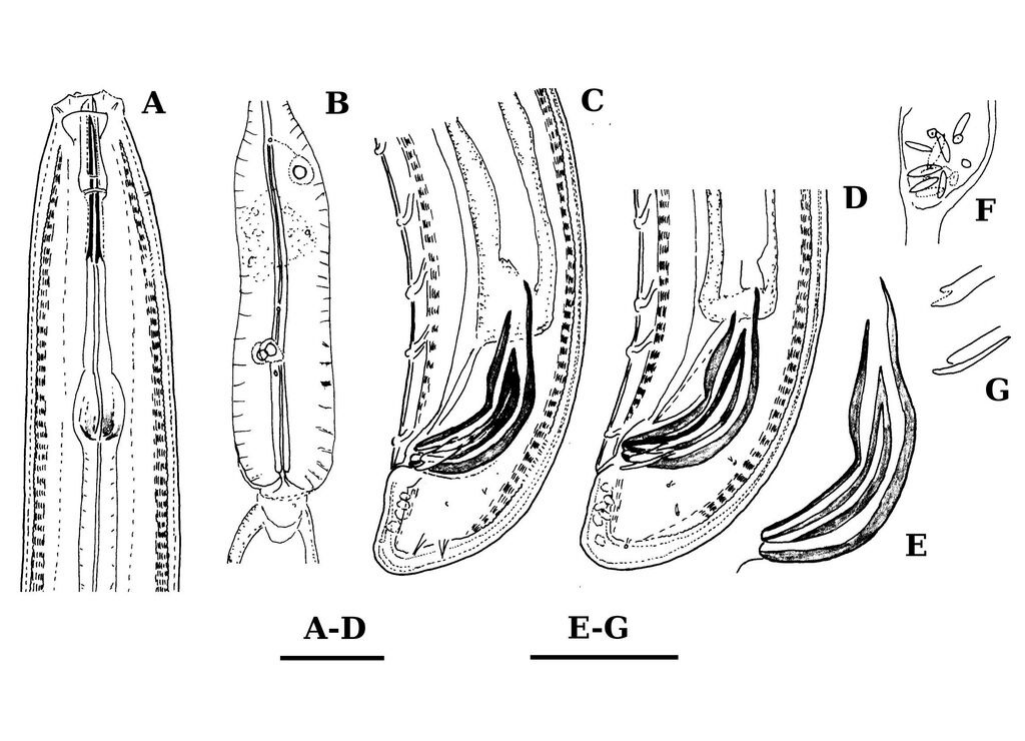
*Enchodelusenguriensis* sp. nov., male. **A** Lip region; **B** Pharyngeal expansion; **C, D** Posterior end; **E** Spicules; **F** Sperm cells; **G** Lateral pieces. Scale bars: **A-D** 25 µm, **E-G** 25 µm.

**Figure 13.**
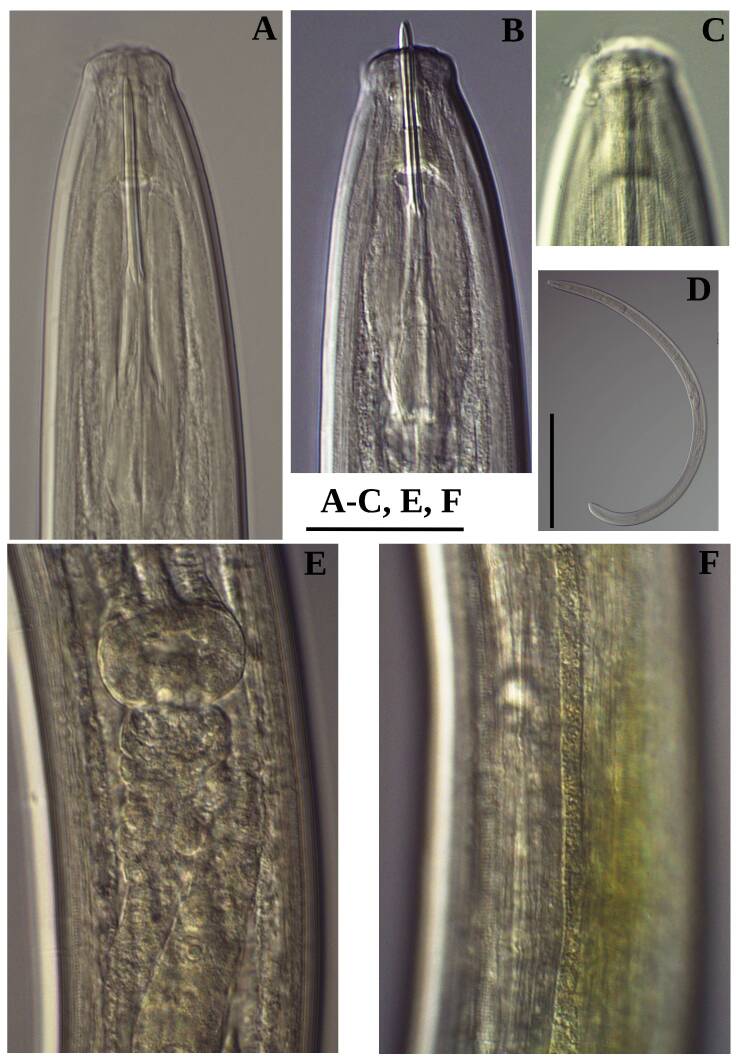
*Enchodelusenguriensis* sp. nov., female. **A, B** Lip region; **C** Amphid; **D** Entire body; **E** *Pars dilatata distalis uteri*; **F** Lateral field. Scale bars: **A-C, E, F** 30 µm **D** 400 µm.

**Figure 14.**
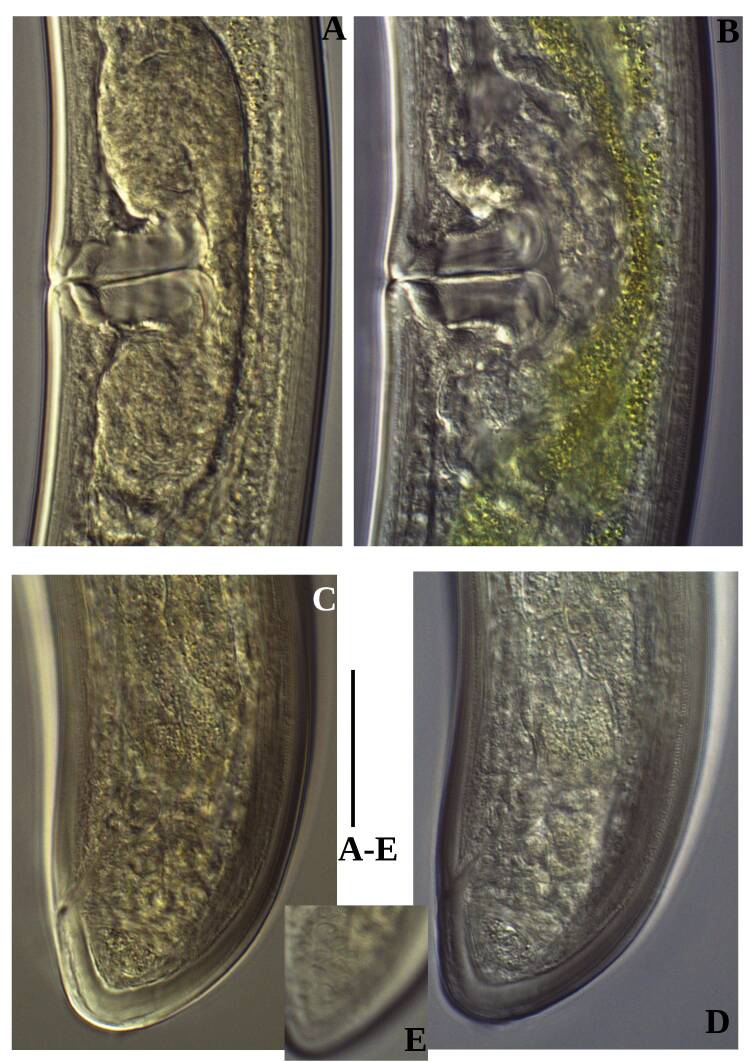
*Enchodelusenguriensis* sp. nov., female. **A, B** Vulval region; **C, D** Tails; **E** Saccate bodies. Scale bar: **A-E** 30 µm.

**Figure 15.**
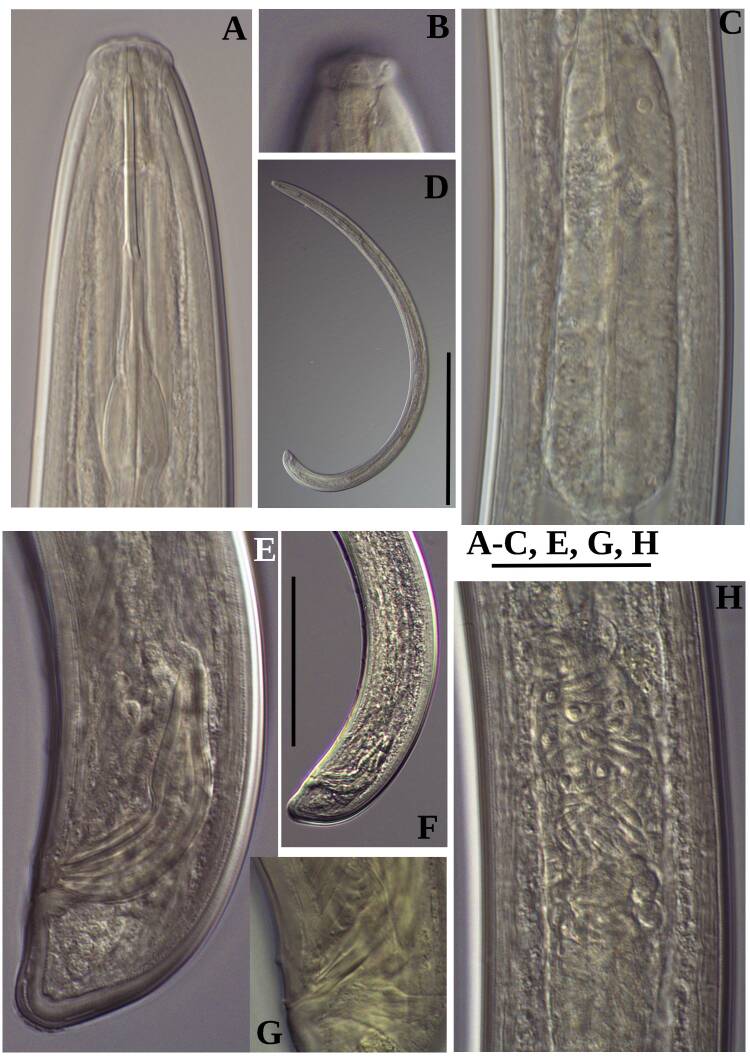
*Enchodelusenguriensis* sp. nov., male. **A** Lip region; **B** Amphid; **C** Pharyngeal expansion, dorsal gland; **D** Entire body; **E** Tail end and spicules; **F** Posterior end; **G** Lateral piece; **H** Sperm cells in testis. Scale bars: **A-C, E, G, H** 30 µm; **D** 400 µm; **F** 100 µm.

**Adult** (Figs 11, 12, 13, 14, 15). Moderately slender (*a* = 20-27) nematodes of medium size, 1.03-1.40 mm long. Body cylindrical, tapering towards the ends. Upon fixation, habitus regularly curved ventrad, C-shaped. Cuticle two-layered, 3-3.5 µm thick at level of the guiding ring, 2.5 µm at mid-body and 6-8 µm on tail, layers with different refraction, the outer one visibly thinner than the inner one and bearing very fine transverse striation, inner layer with slight radial striation, especially visible at tail. Lateral chord very narrow, with granular aspect and lacking any differentiation. Several dorsal and ventral body pores are present at cervical region. Lip region rounded, offset from the adjoining body by a weak, but distinct depression, 2.7-3.6 times as wide as high, with mostly amalgamated lips and distinct papillae visibly protruding above the lip region contour. Amphidial fovea funnel-like, its aperture more than half of lip region diameter. Cheilostom a truncate cone to almost cylindrical, thick-walled. Odontostyle long and slender, straight, 2.1-2.5 times the lip region diameter, 18-20 times as long as wide and 2.6-3.7% of body length, with very narrow lumen and minute aperture occupying hardly 5% of its length. Guiding ring double, located at 1.3-1.7 times lip region diameter from anterior end. Odontophore 1.1-1.2 times as long as odontostyle, thickened, bearing flange-like extensions at its base. Pharynx entirely muscular, gradually enlarging into the basal expansion 87-94 µm long that occupies one-third of the total neck length, pharyngeal gland nuclei and their orifices located as follows: DO = 69-70%, DN = 71-74%, SN = 87-89%, PS1 = 41-48%, PS2 = 40-46%. Nerve ring located at 160 μm from the anterior end. Pharyngo-intestinal junction consisting of a short and rounded cardia and a weak ring-like structure surrounding pharyngeal base.

**Female.** Genital system diovarian, with both branches equally developed, the anterior 250 µm long, the posterior 240 µm (n = 1). Ovaries relatively short, often not reaching the sphincter level. Oviduct with well-developed *pars dilatata* bearing distinct lumen. Sphincter between oviduct and uterus well developed. Uterus long, 126-152 μm or 2.5-2.8 times the corresponding body diameter, tripartite, consisting of a wider proximal portion, a longer and much more slender intermediate section with a narrow lumen and surrounded by cluster of hyaline cells and a well-developed, spheroid, distal *pars dilatata*. Vagina extending inwards to 57-59% of body diameter: *pars proximalis* 19-20 x 18.5-19 μm, *pars refringens* with (in lateral view) two rectangular sclerotised pieces whose combined width is 14-17 μm, *pars distalis* 3-5 μm long. Vulva a transverse slit. Prerectum 3.0-4.1, rectum 0.9-1.1 times anal body diameter length. Tail convex conoid, 1.5-1.9% of body length, with bubble–like or saccate bodies mostly on ventral side, inner core occupying 50-70% of tail length, caudal pores two pairs.

**Male.** General morphology similar to that of female. Genital system diorchic, with opposite testes. Sperm cells spindle-shaped, 8-9 x 2 μm. In addition to the ad-cloacal pair, located at 7-9 μm from the cloacal aperture, there is a series of 9-10 spaced ventromedian supplements, one or two within the range of spicules, thus without hiatus. Spicules dorylaimoid, 1.4–1.9 times the body diameter at level of the cloacal aperture, 4-5 times as long as wide its maximum width: head occupying 20-25% of total length, ventral side bearing prominent hump and hollow, posterior end 6-7 μm wide. Lateral guiding pieces cylindrical with asymmetrical bifurcate end (Figs 12, 15), reaching the spicule terminal tip, measuring 13-15 × 2-3 μm. Tail convex conoid, with two pairs of caudal pores.

**Figure 16.**
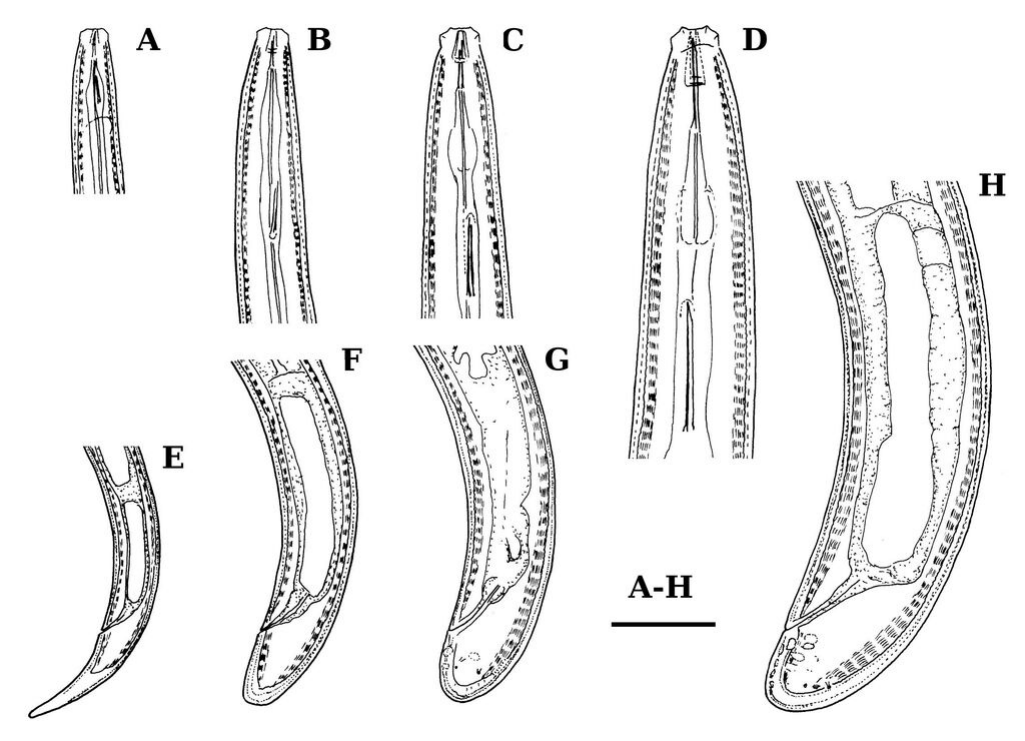
*Enchodelusenguriensis* sp. nov. juveniles. **A-D** Anterior region (A J1, B J2, C J3, D J4); **E-H** Tail ends (E J1, F J2, G J3, H J4). Scale bar: **A-H** 25 µm.

**Figure 17.**
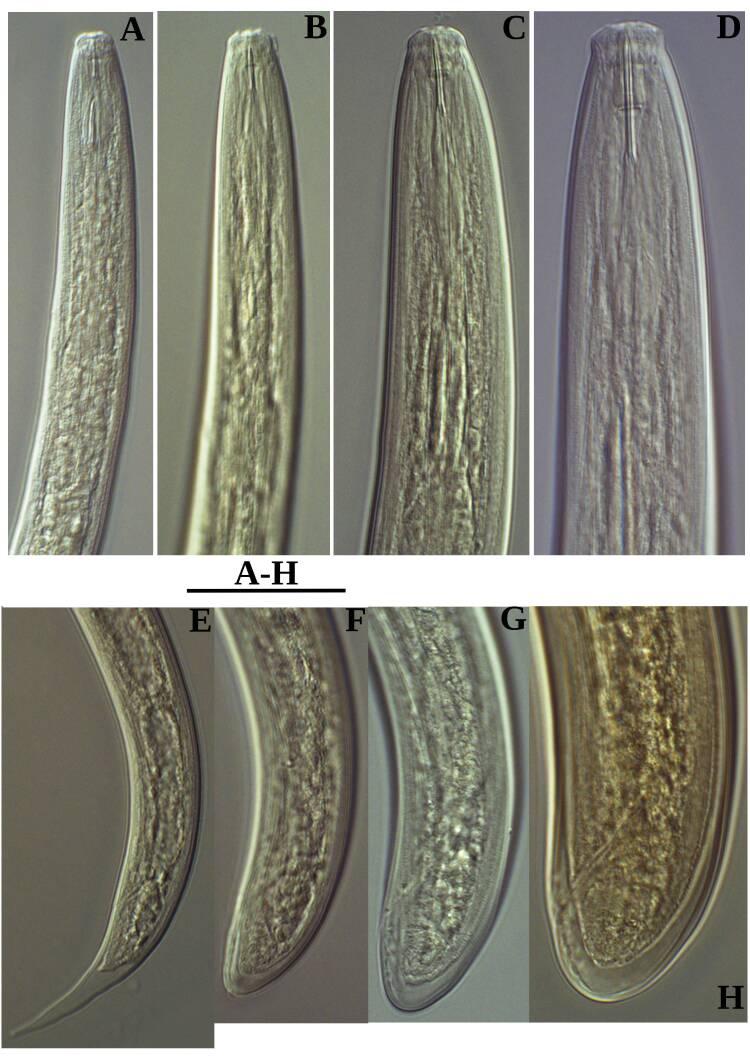
*Enchodelusenguriensis* sp. nov., juveniles. **A-D** Anterior region (A J1, B J2, C J3, D J4); **E-H** Tail ends (E J1, F J2, G J3, H J4). Scale bar: **A-H** 30 µm.

**Juveniles** (*Figs 16, 17*). Based on morphometric of juvenile specimens and the relationships between the lengths of their functional and replacement odontostyles and body lengths, four juvenile stages were identified. Habitus in first juvenile stage almost straight, lip region flat, continuous with the body, genital primordium 8 μm long, tail ventrally curved with long central peg, 20 μm long.

Tail in J2 and J3 conoid elongated in J4 bluntly conoid as in females, c’ decreasing during the successive stages to J4 and females.

##### Molecular characterisation

One D2-D3 of 28S rRNA gene sequences was obtained for this species. In the D2-D3 of 28S rRNA (Fig. 4) gene tree, this sequence formed a highly-supported clade (PP = 100) with *E.macrodorus* and other three *Enchodelus* sequences from GenBank.

#### Diagnosis

*Enchodelusenguriensis* sp. nov. is characterised by its 1.03-1.40 mm long body, lip region 16-17 μm wide and offset by weak depression, odontostyle 37–40 μm long or 2.1-2.5 times lip region diameter, odontophore flanged and 1.1–1.2 times as long as odontostyle, neck 270-290 μm long, pharyngeal expansion occupying one-third of the total neck length, female genital system diovarian, uterus tripartite, *pars refringens vaginae* with two rectangular sclerotisations, vulva (V = 49–53%), tail convex conoid (19-26 μm, c = 51-69, c’ = 0.5–0.8) bearing abundant saccate bodies; males abundant, spicules 54–61 μm long and 9-10 spaced ventromedian supplements without hiatus.

##### Relationships

Based on tail morphology and odontostyle length, this species can be assigned to the *E.macrodorus* – group as defined by Guerrero et al. (2008b). This group includes *E.babakicus*, *E.carpaticus*, *E.distinctus*, *E.groenlandicus*, *E.macrodorus*, *E.makarovae*, *E.microdoroides* and *E.saxifragae*. This homogeneous group is characterised by the presence of a rather long odontostyle (> 35 μm), odontophore with well-developed flanges, uterus tripartite (except for *E.distinctus*, which has been described with a bipartite uterus (Ahmad and Jairajpuri 1980) and hemispheroid to rounded conoid tail.

In having a lip region offset by a depression, the new species is most similar to *E.carpaticus*, *E.groenlandicus*, *E.macrodorus*, *E.makarovae* and *E.microdoroides*. The new species differs from:

*Enchodeluscarpaticus* by its by slightly shorter odontostyle (37-40 vs. 39.5–47 μm), shorter neck length (280-290 vs. 336-388 μm) and pharyngeal expansion (87-94 vs. 136–167 μm), absence of dorsal cell mass near cardia vs. presence, shorter prerectum (90-124 vs. 164–272 mm) and tail (av. 20.5 (19–24) vs. av. 23.7 (21–29) μm), saccate bodies present vs. absent, males present vs. absent (males not found, but sperm cells were observed in one female) (Ciobanu et al. 2010a);

*Enchodelusgroenlandicus* by its shorter body (L = 1.03-1.4 vs. 1.54–2.5 mm) and odontostyle (37–40 vs. 43–53 μm), somewhat more anteriorly located guiding ring (24-26.5 vs. 27–37 μm), narrower lip region (16–16.5 vs. 19–22 μm), more posterior vulva position (V = 49-53 vs. 40-49%), males present vs. absent (Ditlevsen 1927, Andrássy 2009a, Elshishka et al. 2012). A record from Iran (Pedram et al. 2011) largely deviates from the morphometrics of other data for *E.groenlandicus* (e.g. lip region width, tail length) and is not included in the comparison.

*Enchodelusmacrodorus* in having a shorter body (L = 1.03-1.4 vs. 1.38-1.92 mm) and pharyngeal expansion (87-94 vs. 110-145 μm), more posterior vulva position (V = 49-53 vs. 37-47%), a longer and more differentiated uterus (2.5-2.8 times longer than body diameter with long intermediate portion and well developed *pars distalis* vs. 0.9-2.0 times body diam. with very short intermediate region and poorly developed *pars distalis*), tail differently shaped (convex conoid vs. rounded to hemispherical), males abundant vs. males rare (Guerrero et al. 2008b).

*Enchodelusmakarovae* by its shorter body (L = 1.03-1.4 vs. 1.57–2.00 mm), shorter odontophore (1.1-1.2 vs. 1.2–1.4 times as long as odontostyle), shorter neck length and pharyngeal expansion (280-290 vs. 320–377 μm and 87-94 vs. 113–130 μm long, respectively) and uterus (126-152 vs. 220-346 μm), males with shorter spicules (54-61 vs. 65–74 μm long), lateral piece shape (asymmetrically vs. symmetrically bifurcated) (Elshishka et al. 2012);

*Enchodelusmicrodoroides* by having a wider lip region (16–16.5 vs. 13–14 μm), relatively shorter odontostyle (2.3-2.5 vs. 3 times lip region diameter), guiding ring located more anteriorly (24-26.5 vs. 28–39 μm from anterior end), differently-shaped *pars refringens vaginae* (rectangular vs. quadrangular), males abundant vs. males rare, longer spicules (54-61 vs. 45–50 μm) (Baqri and Jairajpuri 1974, Ahmad and Jairajpuri 1980, Brzeski 1992).

The new species can be distinguished from the remaining three species of *E.macrodorus* group by its lip differentiation: lip region set off by depression vs. set off by a deep constriction. Further, it differs from:

*Enchodelusbabakicus* by having cuticle smooth vs. striated, a slightly shorter and thinner odontostyle (37-40 vs. 40-45 μm and 18-20 vs. 13.6-15.3 times as long as wide, respectively), guiding ring located slightly more anterior (24-26.5 vs. 25-30 μm from anterior end), more posteriorly located vulva (V = 49-53 vs. 44-49%), differently-shaped *pars refringens vaginae* (rectangular vs. trapezoidal), thinner hyaline part of tail (29-47 vs. 42-57% of tail length), differently-shaped lateral piece (asymmetrically vs. symmetrically bilobed) (Pedram et al. 2011).

*Enchodelusdistinctus* in having a shorter body length (L = 1.03-1.4 vs. 1.85 mm), longer odontostyle (37–40 vs. 36 μm), more posteriorly located guiding ring (1.5-1.7 vs. 1.4 times lip region diameter), pharyngo-intestinal disc present vs. absent, different structure of uterus (tripartite vs. bipartite), shorter tail (19-24 vs. 32 μm, c’ = 0.6-0.7 vs. c’ = 0.8), saccate bodies present vs. absent, males present vs. absent (Ahmad and Jairajpuri 1980).

*Enchodelussaxifragae* by its shorter body length (L = 1.03-1.4 vs. 1.61-2.38 mm), a narrower lip region (16-16.5 vs. 18–22 μm), shorter neck length and pharyngeal expansion (280-290 vs. 319-490 μm and 87-94 vs. 116–186 μm long, respectively), shorter prerectum (90-124 vs. 140–294 μm) and fewer ventromedian supplements (9-10 vs. 13–16 in number) (Popovici 1995a, Guerrero et al. 2008b, Ciobanu et al. 2010a).

##### Type-locality and habitat

Georgia, Samegrelo-Zemo Svaneti Region, Bogreshi, Enguri River, Tower of Love, moss *Tortellasquarrosa* (Brid.) Limpr., geographical co-ordinates: 43°00’01N 42°50’04”E, 1544 m a.s.l.

##### Type material

The holotype female, three paratype males and four paratype juveniles are deposited in the Nematode Collection of Institute of Biodiversity and Ecosystem Research, Bulgarian Academy of Sciences, Bulgaria, under the accession numbers IBER-BAS NTC 107-109. Other paratypes are deposited as follows: one female, one male and three juveniles (accession numbers ISUZI0010581, ISUZI0010582) – in the Nematode Collection of the Department of Nematology, Institute of Zoology, Ilia State University, Tbilisi, Georgia; one female, one male and three juveniles (accession numbers 0714, 0715) – in the Nematode Collections of the Universidad de Jaén, Jaén, Spain; one female, two males and one juvenile (accession numbers T-8075p, T-8076p) – in the USDA Nematode Collection, Beltsville, Maryland, USA.

#### Etymology

The species is named after the River Enguri; it was recovered from the stone next to the Tower of Love on the bank of the river.

#### Distribution

Georgia

#### Notes

Morphometric data of all species (28) of genus *Enchodelus* are provided in *Suppl. material 1*.

## Supplementary Material

XML Treatment for Enchodelus

XML Treatment for Enchodelus macrodorus

XML Treatment for Enchodelus altherri

XML Treatment for Enchodelus ameliae

XML Treatment for Enchodelus analatus

XML Treatment for Enchodelus arcticus

XML Treatment for Enchodelus babakicus

XML Treatment for Enchodelus brasiliensis

XML Treatment for Enchodelus carpaticus

XML Treatment for Enchodelus decraemerae

XML Treatment for Enchodelus distinctus

XML Treatment for Enchodelus georgensis

XML Treatment for Enchodelus groenlandicus

XML Treatment for Enchodelus hopedoroides

XML Treatment for Enchodelus hopedorus

XML Treatment for Enchodelus laevis

XML Treatment for Enchodelus longispiculus

XML Treatment for Enchodelus lucinensis

XML Treatment for Enchodelus makarovae

XML Treatment for Enchodelus microdoroides

XML Treatment for Enchodelus parahopedoroides

XML Treatment for Enchodelus parateres

XML Treatment for Enchodelus parvus

XML Treatment for Enchodelus ponorensis

XML Treatment for Enchodelus sardashtensis

XML Treatment for Enchodelus saxifragae

XML Treatment for Enchodelus teres

XML Treatment for Enchodelus vestibulifer

XML Treatment for Enchodelus vesuvianus

XML Treatment for Enchodelus enguriensis

BFCBC862-71DF-576C-94F5-8BCC2C46B13F 10.3897/BDJ.12.e126315.suppl1 Supplementary material 1 Morphometric data of the species of genus *Enchodelus* Data type morphometrics File: oo_1033425.xlsx https://binary.pensoft.net/file/1033425 Elshishka M, Mladenov A, Altash S, Álvarez-Ortega S, Peña Santiago R, Peneva V

## Figures and Tables

**Table 1.** Measurements of females, males and juveniles of *Enchodelusenguriensis* sp. nov. from Georgia and *Enchodelusmacrodorus* from Spain. All measurements are in μm (except L in mm) and in the form: mean ± standard deviation with range.

**Character**	***E.enguriensis* sp. nov.**							***E.macrodorus***
	♀ *holotype*	Females (n = 3)	Males (n = 5)	J1 (n = 1)	J2 (n = 2)	J3 (n = 3)	J4 (n = 6)	Female (n = 1)
L	1.4	1.4; 1.2; 1.03	1.3±0.1 (1.2 – 1.4)	0.25	0.45; 0.43	0.52; 0.55; 0.43	0.9±0.1 (0.75-1.02)	1.44
a	25.6	23.3; 23.7; 20.2	26.0±0.9 (24.6-27)	16.0	20.3; 17.0	17.2; 18.3; 14.8	22.1±2.5 (19.2-25.6)	21.1
b	4.8	5.0; 4.2;-	4.6±0.1 (4.5-4.7)	2.1	2.8; 2.7	2.7; 3.4; -	3.6±0.5 (2.9-4.1)	4.4
c	69.1	58.3; 63.6; 54.2	56.6±5.1 (51.3-63.5)	7.7	-; 20.3	27.2; 27.4; 23.9	47.7±6.7 (39.5-57.1)	71.4
c'	0.7	0.6; 0.6; 0.6	0.7±0.1 (0.5-0.8)	2.8	-; 1.2	0.8; 0.9; 0.8	0.7±0.1 (0.5-0.8)	0.5
V%	49	50; 53; 50	-	-	-	-	-	44
Lip region diameter	16	16.5; 16; 16	16.4±0.5 (16-17)	7	9; 9	-; 10; 11	13.1±0.7 (12-13.5)	18.5
Odontostyle length	40	37; 37.5; 38.5	37.1±1.7 (36-40)	7	9; 10	14; 15; 15	25.4±0.9 (24-26.5)	39.0
Replacement odontostyle	-	-	-	8.5	14; 16	23; 23; 24	37.6±0.8 (37-39)	-
Guiding ring	24	-; 25; 26.5	23.2±1.1 (22-25)					25.5
Neck length	285	280; 290; -	283.0±9.7 (270-295)	120	160; 160	190; 160; -	251.3±8.5 (240-260)	326
Body diameter at:								
pharynx base	47	53; 47.5; 47	45.7±1.5 (44-47)	16	22; 24	30; 28; 28	37.6±3.2 (35-43)	60
mid - body	54	60; 51; 51	50.4±2.4 (47.5-53.5)	16	22; 25	30; 30; 29	39.3±3.9 (34-46)	68
anus/cloacal aperture	30	37; 30; 32	35.6±3.6 (31-40)	12	-; 18	23; 22; 24	28.1±1.2 (26-29)	39
Prerectum length	124	-; 90; -	153.9±18.6 (138-174)		-; 60	-; 58; 69	95.8±12.6 (80-110)	185
Rectum length	33	33; 30; 34		10	-; 17	19; 16; -	27.6±2.1 (25-30)	40
Tail length	20	24; 19; 19	23.4±2.9 (19-26)	33	-; 21	19; 20; 18	18.3±2.5 (15-22)	20
Genital primordium	-	-	-	8	14; 15	-; 15; -	38.6±3.6 (34-43)	-
Spicule length	-	-	57.3±2.6 (54-61)	-	-	-	-	-
Ventromedian supplements	-	-	9-10	-	-	-	-	-
